# Temperature and Resource Supply Drive Continental Variation in Size Structure of Freshwater Food Webs

**DOI:** 10.1111/ele.70437

**Published:** 2026-06-24

**Authors:** Vojsava Gjoni, Justin P. F. Pomeranz, James R. Junker, Jeff S. Wesner

**Affiliations:** ^1^ Department of Biology University of South Dakota Vermillion South Dakota USA; ^2^ Institute of Marine Biological Resources and Biotechnology of the National Research Council Mazara del Vallo Italy; ^3^ Department of Physical and Environmental Sciences Colorado Mesa University Grand Junction Colorado USA; ^4^ Department of Biological Sciences University of North Texas Denton Texas USA

**Keywords:** body size, fish, macroinvertebrates, resources, size spectra, temperature

## Abstract

Biological communities follow a remarkably consistent negative relationship between individual mass (M) and abundance (N), represented by a power law (*N* ~ M^λ^). The parameter λ denotes the rate of decline in relative abundance from small to large individuals and serves as a proxy for energy transfer efficiency in food webs. Although warming is expected to affect λ, its influence remains uncertain, possibly due to interactions with resource supply. Using ~670,000 individual body sizes from stream food webs, we tested how temperature and resources shape λ. Temperature effects depended on resource supply (gross primary production [GPP] and organic matter standing stock [OM]) but contradicted expectations that λ becomes more negative with warming. At medium and high resources, λ becomes less negative under warming while no change occurred at low resources. Variation in OM, not GPP, drove these patterns, highlighting the role of external energy inputs and challenging the idea that large organisms are rarer at higher temperatures.

## Graphical abstract

Biological communities exhibit a consistent negative scaling between body size and abundance, yet how this relationship responds to warming remains unclear. Using ~670,000 individual body‐size measurements from stream food webs, we show that temperature effects on size structure depend strongly on resource supply, with warming leading to less negative size spectra at medium and high resource levels but no change under low resources. Variation in organic matter, rather than primary production, primarily drives these patterns, challenging the assumption that warming universally favours smaller organisms.

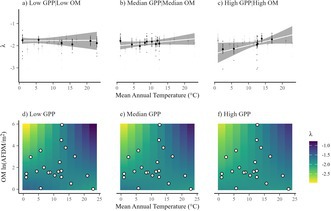

## 1 Introduction

**FIGURE 1:**
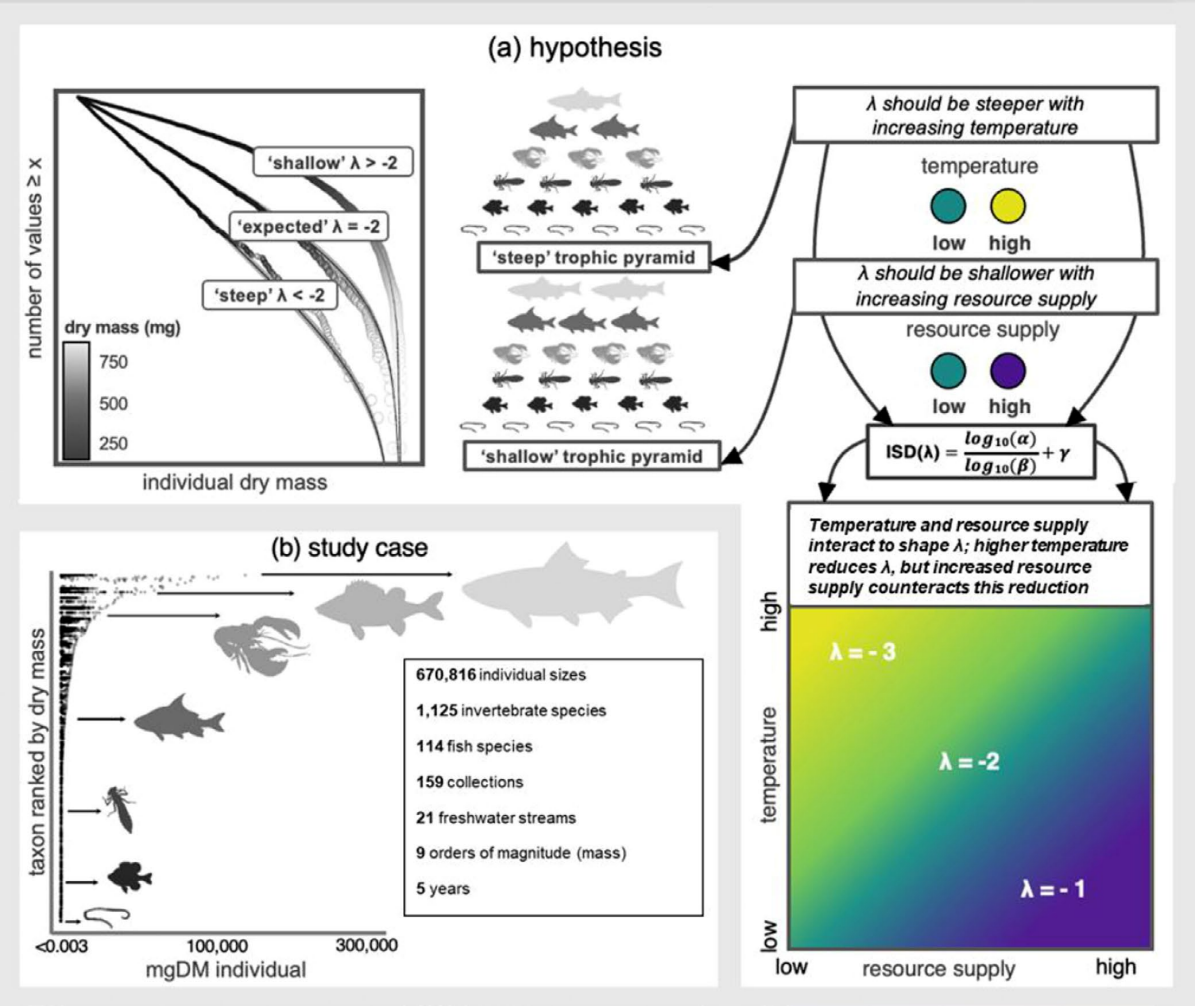
The theoretical basis for Individual Size Distribution (ISD) scaling in ecosystems is underpinned by several empirical studies and theories in ecology. (a) At the community level, λ consistently exhibits a negative pattern with values ~−2. While the individual impacts of temperature and resource supply on the ISD have been extensively investigated, their combined or synergistic effects remain largely unexplored. Our hypothesis is that temperature and resource supply interact to shape λ across food webs. (b) We used NEON continental‐scale data collections (e.g., 670,816 individual sizes), from both fish (114 species) and macroinvertebrates (1125 species). More negative λ values (e.g., < −2), represented by green‐yellow, indicating low resources and high temperatures signify less efficient trophic transfer, supporting relatively fewer large organisms compared to ecosystems with less negative λ values (e.g., > −2), represented by dark purple, indicating high resources and low temperatures.

Earth supports ~550 gigatons of living carbon biomass (Bar‐On et al. 2018; Elhacham et al. 2020) and a fundamental challenge for ecologists is to understand how that biomass is distributed across scales from individuals to ecosystems (Cyr and Pace 1993). The individual size distribution (ISD) is commonly used to describe how biomass is distributed across individuals within communities (Perkins et al. 2019; White et al. 2007) and is driven by the flow of energy through food webs (Blanchard et al. 2017; Petchey and Belgrano 2010). Size‐based assessments, such as the ISD, are emerging as a powerful tool to bridge individual‐level physiological processes to ecosystem‐level patterns (Petchey and Belgrano 2010), complementing more traditional taxonomic or trophic approaches (Blanchard et al. 2017; Brose et al. 2017) in at least two ways: (1) many fundamental aspects of an individual organism's biology are controlled by body size, including metabolic rate, life history, diet breadth and trophic position (Brown et al. 2004; Glazier and Gjoni 2024; White et al. 2007; Woodward et al. 2005) and (2) predator–prey interactions occur between *individual*s, not species per se and as such size‐based approaches may better capture complex trophic relationships such as trophic omnivory (Thompson et al. 2007) intra‐guild predation (Arim and Marquet 2004) and cannibalism that are often overlooked in traditional taxonomic approaches. Therefore, variation in the ISD reflects fundamental changes in the attributes of a community, providing an integrative measure of variability in ecosystem structure and function (Figure 1; Blanchard et al. 2017; Edwards et al. 2017; O'Gorman et al. 2017).

The ISD is described by a power law, *N ~ M* ^λ^, where *N* is abundance and *M* is individual body mass. More formally, because individual masses are continuous measures, the ISD simplifies to a frequency distribution (White et al. 2007): *f(M) ~ M* ^λ^. Metabolic scaling theory predicts that the exponent λ is generated by interactions of three ecosystem‐level variables: trophic transfer efficiency (α), predator–prey mass ratio (β) and metabolic scaling with mass (γ; Dickie et al. 1987; Jonsson et al. 2005; O'Gorman et al. 2017; Reuman et al. 2008; such that: Reuman et al. 2008).

(1) $$\lambda =\frac{\log_{10}\alpha }{\log_{10}\beta }-\gamma –1$$

Given typical values of α, β and γ, the exponent λ tends to be negative, typically around −2 (Figure 1), underscoring a remarkably consistent ecological pattern across Earth's diverse ecosystems (Blanchard et al. 2017; Hatton et al. 2021). Owing to the apparent consistency of the ISD, body size distributions have been suggested as a ‘universal indicator’ of ecological status (Enquist et al. 2024; Petchey and Belgrano 2010). Consequently, ecologists have increasingly used it to indicate fundamental changes in community structure and ecosystem function in response to anthropogenic impacts, including over‐fishing, species invasion and environmental pollution (Blanchard et al. 2017; Hatton et al. 2021; Novak et al. 2024).

Experimental and field‐based results have also shown that λ varies in response to temperature, though the magnitude and direction of change is inconsistent. Increasing temperature is predicted to favour smaller organisms (Daufresne et al. 2009) leading to more negative values of λ (i.e., ‘steeper’ slopes) and this has been observed empirically. However, warming can also favour larger organisms or have no response (Mazurkiewicz et al. 2020). One explanation for the inconsistent response of size‐abundance scaling to temperature is through compensatory effects of resource supply at the base of the food web (O'Gorman et al. 2017). Increasing temperature induces higher metabolic costs, leading to reduced efficiency of trophic transfer and a subsequent selection for smaller body sizes. But these effects can be counteracted by increases in resource supply, which can fulfil increased energetic demands, especially for larger individuals under warmer temperatures (Brown et al. 2004; Junker et al. 2020). However, how temperature and resources interact to affect λ in food webs is rarely studied (Cross et al. 2015).

Testing how λ responds to temperature and resource supply at the macroecological scale is logistically challenging because it requires data‐intensive measures of individual body sizes, accurate (daily or sub daily) temperature measures and estimates of resource supply (gross primary production, allochthonous subsidies; O'Gorman et al. 2017; Perkins et al. 2018). Additionally, a key assumption in ecological scaling laws is that communities operate under demographic and resource steady‐state conditions (West et al. 2009). This means that, on average, resource consumption balances supply, birth rates match mortality and a stable size structure is maintained. As a result, single‐point measurements of ISD may only capture temporary deviations caused by recent disturbances, rather than reflecting long‐term ecological patterns. To understand these dynamics, large‐scale long‐term monitoring efforts are essential.

Here, we overcome these challenges by using data from the National Ecological Observatory Network (NEON). We examined the spatial and temporal responses of 159 ISD's using individual fish and macroinvertebrate body sizes collected from 21 freshwater streams in North America. We hypothesized that λ would decline with temperature overall, due to increased metabolic demand at high temperatures, but that the magnitude of the decline would become weaker as resource supply increased. We further hypothesized that the interactive effect of temperature and resource supply on λ would be consistent across years, which we tested using hierarchical structure in the model with varying intercepts and slopes by year. Because NEON sampling spans broad spatial and temporal gradients, repeated samples within sites capture variation around a long‐term mean size structure rather than short‐term variability.

## 2 Materials and Methods

### 2.1 Body Size Data

**FIGURE 2:**
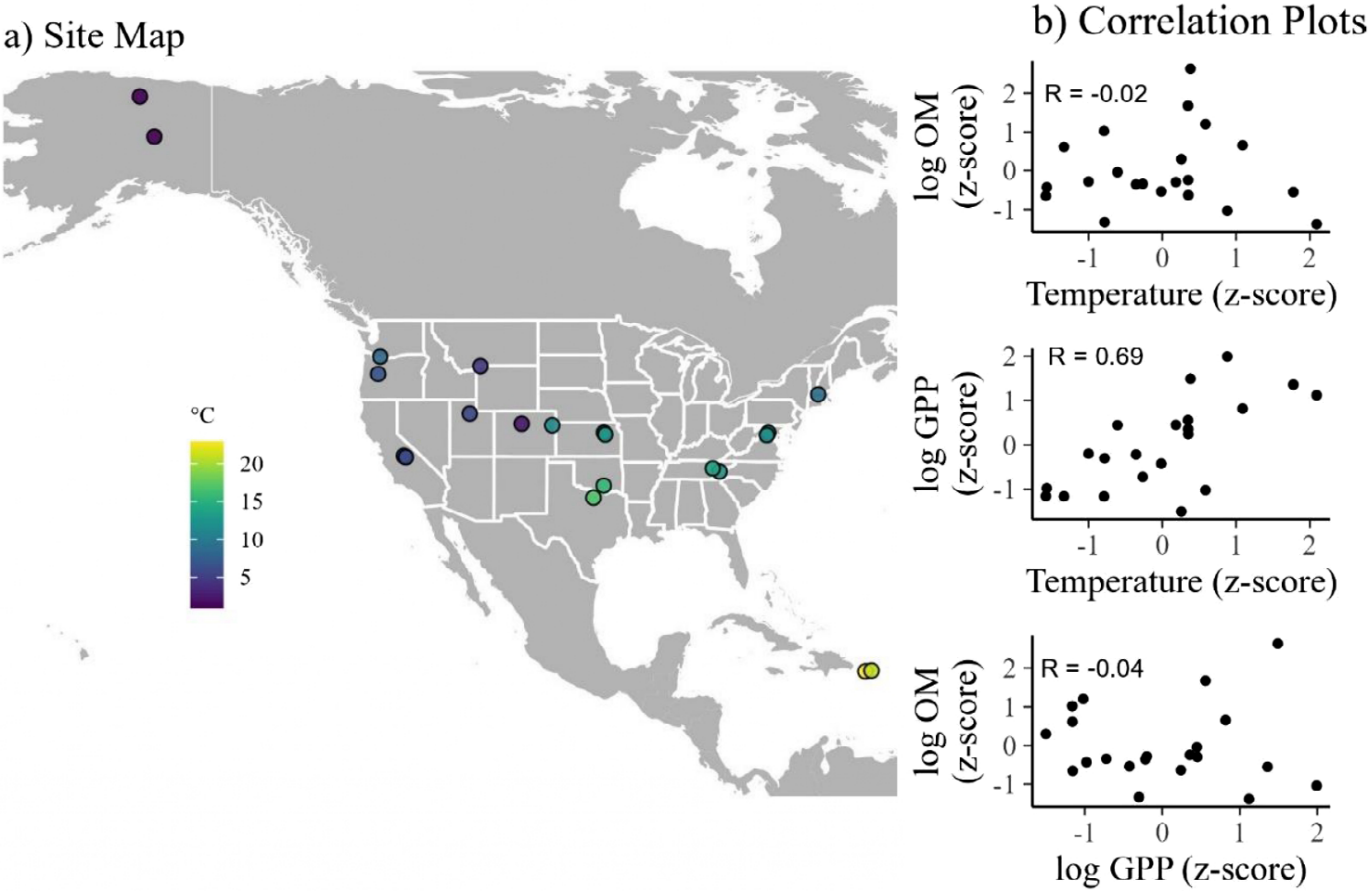
Variation in temperature and correlations of temperature, gross primary production (GPP) and organic matter (OM) across NEON stream sites (*n* = 22). (a) NEON sampling sites span a wide range of mean annual temperatures (1°C to 24°C). Each sampling site is a point on the map, with temperature reflected by colour. (b) Weak correlations in environmental variables. Values for temperature are standardized annual means. Values for GPP are standardized natural log‐transformed annual means. Values for organic matter represent standardized natural log‐transformed standing stocks averaged across 2–4 samples per site. ‘log’ is the natural log. Standardized values represent z‐scores.

We obtained 670,816 individual body masses of fish and macroinvertebrates collected by the National Ecological Observatory Network (NEON) from 2016 to 2022. NEON is a continental‐scale ecological sampling program that uses standardized, automated sensor measurements coupled with observational field data and biological collections repeated across seasons (spring, summer, autumn), years (2015 to present) and space (across North America; Figure 2). The final data set included 59,083 measures from 114 fish species and 611,733 measures from 1125 macroinvertebrates taxonomic groups (typically genus or species). The samples were typically collected > 1 time per year at each of 21 sites, resulting in a total of 159 unique collection events. Below we describe how the data sets were merged to contain both fish and macroinvertebrates. A schematic overview of this workflow is in Figure S1.

#### 2.1.1 Macroinvertebrates

NEON collected macroinvertebrate data via fixed‐area samplers (e.g., Surber/Core/Kicknet) and measured insect body lengths to the nearest mm along with estimates of their density (individuals m^−2^). While the samplers vary based on the stream characteristics (e.g., core samplers, Surber samplers, etc.), all mesh sizes are the same (243 μm). Measurements of macroinvertebrate lengths spanned from 1 to 86 mm. We converted them to dry mass (mg) with published taxon‐specific length‐mass regressions (Gjoni et al. 2019; Pomeranz et al. 2022).

#### 2.1.2 Fish

Fish were collected from each site twice per year (typically) using 3‐pass removal electrofishing. For each collection, the first 50 fish per taxon were measured for wet mass (wm) in g (NEON 2022). We converted this to dry mass (dm) using the equation dm=0.2wm (Brey et al. 2010). The remaining fish were tallied as a bulk count per species (without individual mass measures). Because individual body masses were measured only for a subset of individuals, raw measurements alone would not reflect ‘true’ species abundances, particularly when total abundance varied widely among taxa. Using the three‐pass depletion data, we estimated fish population density (individuals m^−2^) in each collection using a multinomial Poisson depletion model (Royle et al. 2004). We specified the model in R using the *ubms* package (Kellner et al. 2022). The response variable was the number of fish caught per pass and the predictor variable was the collection id (*site* + *date* + *reach* = collection id). The model resulted in a population estimate for each collection, which we converted to individuals m^−2^ by dividing each estimate by the sampled area (mean stream width × length of the electrofishing sample). We then multiplied that population estimate by the relative abundance of each fish body mass, resulting in an estimated density (individuals m^−2^) of each fish body mass in each collection.

#### 2.1.3 Combining Fish and Macroinvertebrates

Fish and macroinvertebrates were collected on different dates, with macroinvertebrates collected three times per year and fish collected twice. Therefore, to combine fish and macroinvertebrate samples, we limited the data to only collections that occurred within 30 days of each other. For example, if macroinvertebrates were collected on June 10 and fish collected on June 20, those samples were combined. If more than one sample was in the 30‐day window (e.g., another fish collection on June 21), we selected the samples collected closest to each other in time. The resulting data set contains body sizes ranging nine orders of magnitude (0.003 to 200,000 mg dry mass) along with their densities. Some body sizes were the same for fish and macroinvertebrates but had different densities. In such cases, we averaged the density of each body size so that each row contains a unique body size. We used this dataset to estimate individual size distributions (see Data Analysis).

### 2.2 Environmental Data

#### 2.2.1 Temperature

To estimate mean annual stream temperature for each of the 21 sites, we obtained water temperature readings collected every 4 h from 2016 to 2021 using the NEON data (Table S1). We removed data that did not pass NEON quality control checks. Specifically, we removed data that appeared unreasonably such as low (< −5°C) or high (> 50°C) temperatures. Some data for Alaskan streams are missing when the water is frozen. For those data, we assumed a temperature of 0°C. The resulting dataset contained 93,930 temperature readings. Temperature was modelled using a Bayesian generalized additive mixed model (GAMM), with a smooth term for temperature and year included as a random effect. Although the model has a generalized additive structure, it was fit within a Bayesian framework, allowing direct estimation of posterior distributions for the temperature effect. To reduce the data size for modelling, we estimated the mean weekly temperature and modelled that as a function of date and site using a Bayesian generalized additive mixed model with a Gaussian likelihood and year as a varying intercept. Mean weekly temperature was centred prior to modelling. This approach allowed us to have a posterior distribution of temperature predictions on each day over 3 years. From that posterior distribution, we calculated the mean annual temperature and standard deviation (Table 1) for each site.

**TABLE 1:** Mean annual (± SD) values of temperature (°C) and gross primary production (GPP, g C m^−2^ year^−1^), along with standing stock organic matter (OM, g AFDM m^−2^), at the 21 NEON sites in this study.

Site	Temperature	GPP	OM
ARIK	12 ± 8	1728 ± 396	5 ± 4
BIGC	10 ± 5	727 ± 194	4 ± 2
CARI	1 ± 2	353 ± 44	3 ± 1
GUIL	21 ± 2	4235 ± 1573	3 ± 4
HOPB	9 ± 7	541 ± 198	5 ± 5
KING	12 ± 5	1919 ± 580	92 ± 126
LECO	13 ± 5	1402 ± 387	3 ± 1
MART	8 ± 4	891 ± 312	5 ± 1
OKSR	1 ± 3	420 ± 256	4 ± 2
REDB	6 ± 4	821 ± 326	1 ± 2
WALK	14 ± 3	403 ± 40	46
WLOU	2 ± 3	351 ± 88	19 ± 7
LEWI	13 ± 4	4828 ± 823	377 ± 418
TECR	6 ± 5	350 ± 182	35 ± 21
BLUE	16 ± 4	7925 ± 2536	2 ± 0
CUPE	23 ± 1	3344 ± 298	1 ± 1
MCDI	13 ± 7	1573 ± 1205	5 ± 8
MCRA	7 ± 4	1719 ± 308	7 ± 4
POSE	12 ± 7	250 ± 48	12 ± 13
PRIN	17 ± 7	2479 ± 557	20 ± 23
BLDE	4 ± 5	907 ± 270	5 ± 4

*Note:* Values are averaged over multiple years for temperature and GPP using sensor readings from NEON. For OM, values are calculated directly from benthic samples.

#### 2.2.2 Gross Primary Production

We estimated regimes of annual gross primary production (GPP, g C m^−2^ year^−1^) through a multistep ensemble model process (https://zenodo.org/records/20704567). Daily time series of NEON sensor‐based data for temperature, stream discharge and oxygen concentration (see Table S1) were checked for data quality flags and visually inspected to identify potential errant data. We then estimated daily GPP from cleaned time series with maximum likelihood estimation through 15 different methods using the ‘*streamMetabolizer*’ package (Appling et al. 2018). Methods varied in respect to discharge–gas exchange, photosynthetic rate–light and photosynthetic rate–temperature relationships. From this model suite, we first calculated root mean squared error (RMSE) based on pointwise differences between predicted and observed daily oxygen series. This model suite was reduced by removing models that yielded unreasonably high estimates based on mean daily GPP (> 13,000 g C m^−2^ year^−1^), correlation coefficients > 0.95 between reaeration and ecosystem respiration, and particularly poor fits based on RMSE. From this filtered model suite, we created an ensemble model weighted by relative RMSE. Finally, to develop a general model of the GPP regime at each site, we fit a hierarchical generalized additive model (GAMM) to ensemble estimated GPP by ‘day of year’ with ‘year’ treated as a random effect term. We summed the estimated ‘day of year’ fixed effect to quantify annual GPP for all sites. Annual GAMMs were fit with the ‘*brms*’ package (Bürkner 2017) with smoothing functions from the ‘*mgcv*’ package (Wood 2017).

#### 2.2.3 Organic Matter

We estimated the standing stock of organic matter from 45 unsorted bulk benthic samples in the NEON biorepository (at least two samples per site; Yule and Franz 2020). The samples were collected at the same time as macroinvertebrate samples, using the same collection techniques (i.e., Surber sampler, core sampler). We obtained the raw samples from NEON and first removed macroinvertebrates. We dried the remaining organic matter at 60°C for > 48 h to bring to a constant mass. We then combusted samples at 500°C for 4 h before reweighing to determine total organic matter mass. Samples were scaled to areal mass (g·m^−2^) by dividing total organic mass by the benthic area sampled (e.g., the area of the Surber sampler, core sampler, etc.).

### 2.3 Data Analysis

To examine how the ISD varied as a function of temperature and resources, we used a Bayesian generalized linear mixed model with a truncated Pareto likelihood. A description and justification of this modelling approach for ISD's is given in Wesner et al. (2024). The model structure was:

$$x_{\text{ijkl}}\sim f\left(x_{\mathrm{jkl}};\lambda_{\mathrm{jkl}},x_{\min_{\mathrm{jkl}}},x_{\max_{\mathrm{jkl}}},{\text{counts}}_{\text{ijkl}}\right)$$

$$\lambda_{\mathrm{jkl}}=a+\mathrm{\beta Z}+\gamma_{k:j}+\gamma_{l}+b_{l}Z$$

where $x_{\text{ijkl}}$ is the *i* ^th^ body size from sample *j* in site *k* and year *l*. The likelihood *f(…)* is a truncated Pareto with a single free parameter $\;\lambda_{\mathrm{jkl}}$, the exponent of the ISD. $x_{\min_{\mathrm{jkl}}}$ and $x_{\max_{\mathrm{jkl}}}$ are the minimum and maximum body sizes in each sample, site and year. $x_{\min_{\mathrm{jkl}}}$ was chosen as the smallest body size in each sample for which the data most closely match a power law using the *estimate_xmin* function from the poweRlaw package (Gillespie 2024). This method ensures that estimates of λ are not biased from undersampling of small individuals (Virkar and Clauset 2014). After removing undersampled sizes, 228,748 of the original 670,816 (34%) were retained for analysing the ISD. Each body size has a corresponding density in units of individuals per m^2^, represented by *counts*_*ijkl*_, as described in Edwards et al. (2017). $\lambda_{\mathrm{jkl}}$ is modelled as a linear function of an intercept α and βZ represents the single, two and three‐way interactions of the **Z** predictors of mean annual temperature, mean annual GPP and standing stock organic matter. All predictors were standardized as z‐scores prior to fitting. Varying intercepts are included for individual sample *j* nested within sites *k* $\left(\gamma_{k:j}\right)$ and year $\left(\gamma_{l}\right)$ along with varying slopes, including interactions, by year $b_{l}Z$. To improve sampling efficiency, the varying intercepts were modelled using non‐centred parameterisation, which is excluded here for clarity, but is present in the Stan model code: https://zenodo.org/records/20704567.

Priors for the intercept were Normal (−2, 0.5), chosen based on theoretical predictions (Reuman et al. 2008) and previous analysis of ISD values in streams (Pomeranz et al. 2022). Priors for each *β* parameter were set to Normal (0, 0.2). Priors for the *q* varying intercepts were Normal (0, s_q_), with each s_q_ hyperprior set to Exponential (7). These priors were chosen based on prior predictive simulation (Wesner and Pomeranz 2023) to centre the prior probabilities of λ between about −2.5 to −1 while still allowing probabilities at very large (e.g., 1) or small values (e.g., −4; Figure S3).

We fit all models in ‘*rstan’* (Stan Development Team 2024) via the ‘*brms’* (Bürkner 2017) and ‘*isdbayes’* (Wesner and Pomeranz 2023) packages in R. Each model had 4 chains with 2000 iterations, where the first 1000 were discarded as warm‐up. Model fit was checked using posterior predictive checks and prior influence was checked using prior predictive simulation and prior sensitivity analysis (Figures S3–S5). To further illustrate model fit, we provide example plots of observed ISDs and fitted truncated Pareto distributions for 21 randomly selected collection events in the Figure S6. For model checking, it is essential that the data all have equal *counts*_*ijkl*_. Therefore, prior to model checking, we resampled the dry mass measures with replacement, weighted by *counts*_*ijkl*_. An example of this procedure is given in Figure S2.

To assess the robustness of our results to seasonality, taxonomic composition (e.g., fish versus macroinvertebrates), macroinvertebrate sampling method and spatial autocorrelation, we conducted a series of sensitivity analyses in which we re‐fit the model described above five times (see Supporting Information S1). First, we re‐fit the model using only the first samples collected per site within each year. Second, we re‐fit the model using only the last samples collected per site within each year. Comparing these two models to the full model above allowed us to evaluate whether inference depended on the timing of sampling collection within a year, without imposing a categorical seasonal definition across a broad latitudinal gradient. Third, we re‐fit the model using only macroinvertebrate body sizes or only fish body sizes to assess the influence of taxonomic composition. Fourth, we re‐fit the model using only macroinvertebrate data that were collected with Surber samplers to evaluate potential effects of sampling methods. Fifth, to assess spatial autocorrelation, we re‐fit the model including a Gaussian process‐based varying intercept structured by site latitude and longitude and compared the outcomes to the full model. Finally, to place our results into context of previous work, we extracted effect sizes of previous studies that have measured λ responses to temperature (summarized in Pomeranz et al. 2022) and overlaid them with the mean temperature effect estimated in this study.

## 3 Results

### 3.1 Temperature and Resource Supply Interact to Shape λ

**FIGURE 3:**
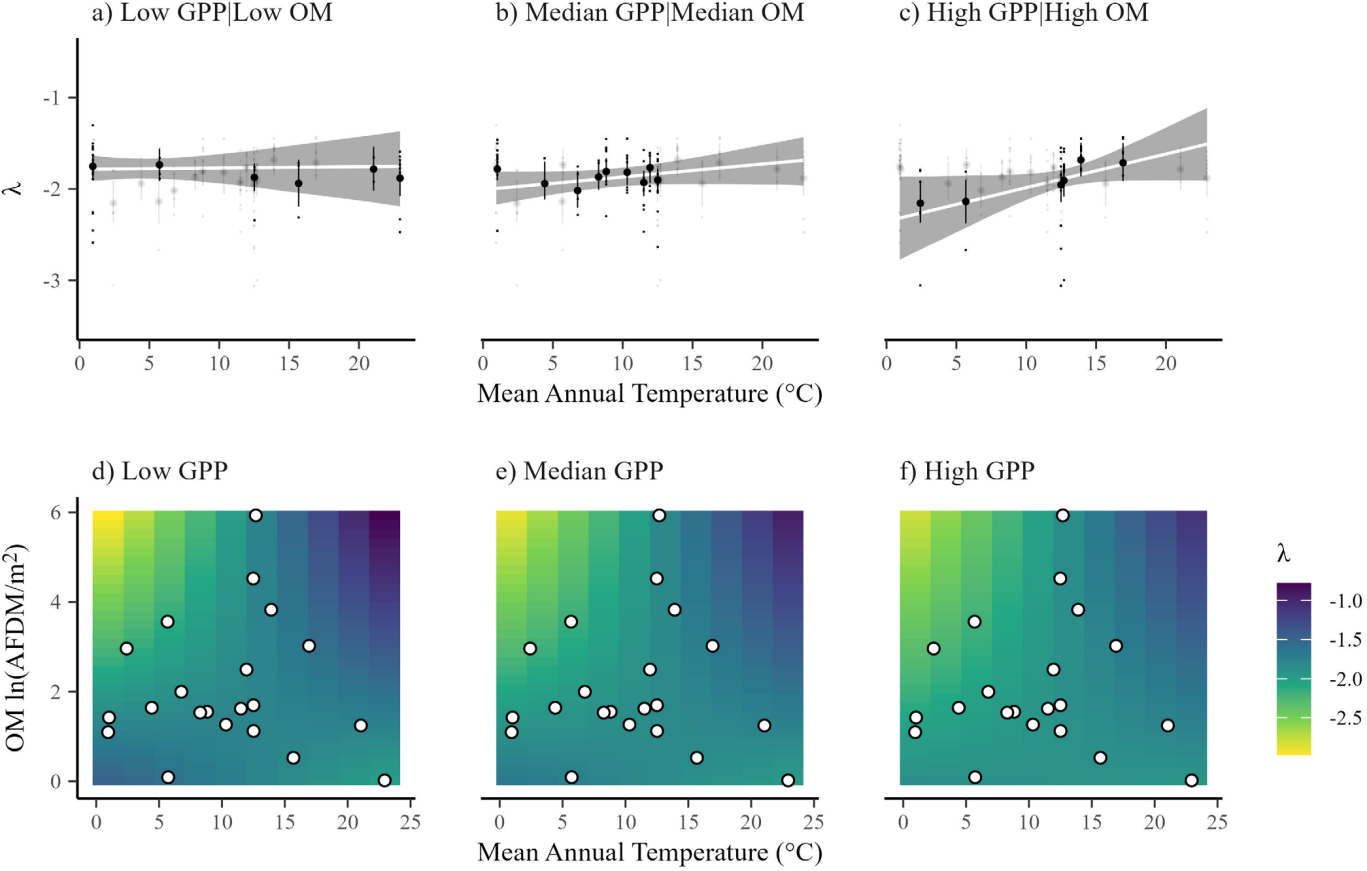
Relationships of the ISD across mean annual stream temperature, annual gross primary production (GPP) and organic matter (OM). (a–c) The relationship between temperature and λ depends on resource supply. At low resource levels [25th percentiles of ln(OM z‐scores) and ln(GPP z‐scores)], the relationship is flat but is positive at median (50th percentiles) and high resource levels (75th percentiles). The small dots are individual sample λs (*n* = 159). Points corresponding to samples within the low, median and high resource categories shown in each panel are highlighted to indicate the observations most relevant to each fitted relationship, while all other samples are partially transparent. The larger dots and error bars are posterior medians and 95% CrI for each year and site combination. For organic matter, low and high percentiles correspond to approximately 3 and 20 g AFDM m^−2^, respectively, which are well below the most extreme observed values across sites (92 and 377 g AFDM m^−2^). For GPP, low and high percentiles correspond to approximately 420 and 1900 g C m^−2^ year^−1^, again well below the maximum observed across NEON sites (~7000 g C m^−2^ year^−1^). (d–f) Heat map showing model predictions of λ (posterior medians) across combinations of mean annual temperature and organic matter (OM) at low (d), median (e) and high (f) levels of gross primary production (as described above). The white dots show the observed temperature, OM and GPP of each stream (*n* = 21). While the model predicts high (e.g., λ ~ −1, purple) and low (λ ~ −2.5, yellow) values across the full environmental space, the three heat maps appear visually similar because, within the observed range of GPP, variation in λ is driven primarily by temperature and OM, not GPP.

Across 159 samples, λ ranged from −3 to −1.2 (posterior medians) with an average of −1.9 (Figure 3a; 95% credibility interval [CrI]: −1.99 to −1.8). Averaging across these samples and years for individual streams revealed stream medians ranging from −2.1 to −1.6. In support of our hypothesis, λ varied with temperature and the direction of change depended on resource supply. However, in contrast to theory temperature had a primarily positive or neutral effect on λ, meaning that warmer streams supported a higher proportion of large individuals than expected. At low levels of resource supply (i.e., 25th percentile of OM and GPP), λ did not change with increasing temperatures [standardized slope = −0.01 (−0.12 to 0.14)] (Figure 3). This pattern shifted as resource supply increased. At median levels of resource supply, λ became less negative with increasing temperatures [0.09 (0.01 to 0.20)] with a 96% probability of a positive slope (Figure 3). At high levels of resource supply (i.e., 75th percentile), the slope was also positive but more uncertain [0.23 (0.01 to 0.45), 99% probability of positive] (Figure 3).

Among the resource supply types, variation in organic matter standing stock accounted for most of the influence of resource supply, rather than gross primary production. For example, when gross primary production is held constant, the slope between temperature and λ changes from flat at low OM (0.01 [−0.12 to 0.14]) to strongly positive at high OM (0.22 [0.07 to 0.38]; Figure S7), a 22‐fold increase. By comparison, there is minimal change in the temperature effect across levels of GPP when OM is held constant, with the slope at high GPP (0.12 [−0.01 to 0.27]) only 2‐fold higher than at low GPP (0.06 [−0.05 to 0.17]; Figure S7).

Across the range of stream temperatures, our model predicted λ to increase from −2.1 (−2.3 to −1.9) at cold streams near 0°C to −1.6°C (−1.9 to −1.4) at warm streams near 25°C (assuming median levels of resource supply), a difference of 0.5 units (Figure 3). While the absolute units appear small, the implications for changes in the food web are potentially large. For example, using the probability density function of the truncated Pareto distribution, we calculated the probability of sampling a given body size under different λ values. When λ = −1.6, a 10 g (dry mass) individual occurs with a probability of 0.5%. When λ = −2.1, the same individual occurs with a probability of 0.01%, an order of magnitude decline that suggests a 10 g individual is 50 times more likely at the site with λ = −1.6. By comparison, a 0.001 g individual is only 1.75 times more likely. This sizeable change in the probability of large individuals underscores potentially substantial changes in the energetic demand and transfer within food webs.

### 3.2 Effects Are Consistent Across Years

To determine how the results above varied temporally, we used the varying slopes and intercepts to estimate relationships between temperature and λ across years. The positive or neutral relationships between temperature and λ (when resources are at their median) were consistent in all years (Figure S8), with slopes ranging from 0.07 to 0.12 (in 2021 and 2020, respectively) with 95% CrI all including the global mean slope of 0.14. Similarly, the interactions of resource supply and temperature were also consistent across years (Figure S9).

### 3.3 Robustness of Results to Seasonality, Taxonomy, Sampler Type and Spatial Autocorrelation

Effects were generally robust to seasons, taxonomic groupings, sampler type and spatial autocorrelation. Re‐fitting the models using only samples collected early or late within each year produced patterns consistent with the main results described above, including no effect of temperature on λ at low resource levels and a weak positive effect (~77% probability of a positive slope) at higher resource levels (Figure S10). Accounting for spatial autocorrelation using Gaussian process‐based random effects also produced similar results, with stronger evidence of a positive temperature effect at high resource levels (97% probability of a positive slope; Figure S10). Furthermore, analyses that were limited to macroinvertebrates or to macroinvertebrates from only Surber samplers (along with fish) revealed similar responses (Figure S10). Analyses conducted only with fish differed from the above results; the temperature effect was similar under all resource scenarios (Figure S10) with the strongest evidence of a positive slope at moderate resource levels (91% probability of a positive slope). Most importantly, none of the data combinations we tested revealed evidence for a negative effect of temperature on λ. Together, these results indicate that the observed temperature–resource interactions are robust to seasonal sampling differences, taxonomic composition and spatial autocorrelation.

## 4 Discussion

The most important result of this work is that streams supported a higher fraction of large individuals in warmer compared to colder environments, a pattern opposite of that predicted by theory and much of the literature (though not universally). Across streams, empirical estimates of λ clustered near the canonical theoretical expectation of −2, but were consistently slightly less negative (posterior median = −1.9; 95% CrI: −1.99 to −1.8), indicating a relatively higher contribution of large individuals than expected. Interpreting this result requires accounting for the hierarchical and spatial structure of the dataset: because sampling sites overlap spatially and include repeated observations within sites, the observed responses reflect variation in site‐level size‐structure along environmental gradients rather than discrete spatial differences among locations.

**FIGURE 4:**
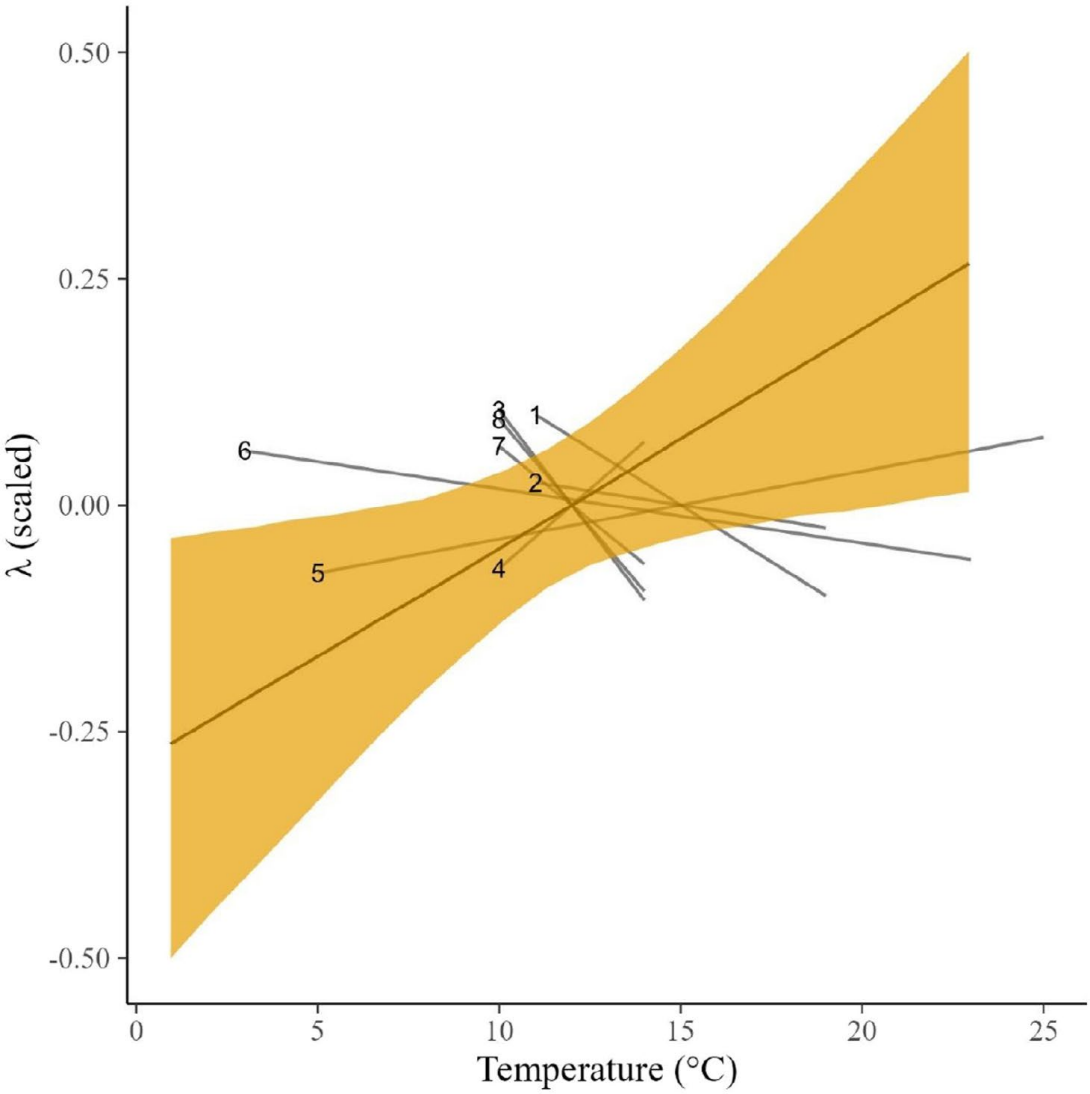
Variation in literature estimates of the ISD‐temperature relationship compared to the current work. The current NEON‐based analysis is in orange. Individual lines show the change in lambda across 9 additional studies. Lambda on the y‐axis is centred to the mean for each study to make the different approaches in reporting and estimating lambda comparable. The figure shows several large temperature ranges (~15°C), with most studies having relatively narrow ranges (~5°C), with particular focus on temperate streams around 12°C. Lambda values were estimated from figures, tables, or summary statistics in the corresponding study: One (Arranz et al. 2023a), two (Arranz et al. 2023b), three (Dossena et al. 2012; April estimate), four (Dossena et al. 2012; October estimate), five (O'Gorman et al. 2017), six (Pomeranz et al. 2022), seven (Yvon‐Durocher et al. 2011; community), eight (Yvon‐Durocher et al. 2011; phytoplankton only). Our findings highlight how resource supply mediates the temperature effect on λ, explaining variability overlooked in earlier work.

Viewed in this context, our findings extend prior studies that have tested temperature effects on λ within individual taxonomic groups (e.g., phytoplankton; Yvon‐Durocher et al. 2011), macroinvertebrates (Pomeranz et al. 2022), fish (Arranz et al. 2023a) or across relative narrow size or temperature ranges (Arranz et al. 2023a). These studies are often spatially and temporally constrained (Dossena et al. 2012; O'Gorman et al. 2017; Yvon‐Durocher et al. 2011), exhibit seasonally dependent patterns (e.g., April vs. October) and typically span limited temperature gradients, ranging from ~4°C (Arranz et al. 2023a; Dossena et al. 2012; Yvon‐Durocher et al. 2011) to ~15°C (Pomeranz et al. 2022). To place this variation in a broader context, we overlaid the standardized temperature effect sizes from the literature with the mean temperature effect estimated in this study (Figure 4). Although reported effects of temperature on λ vary among studies, effect sizes are generally small and largely overlap with the uncertainty in the present study (Pomeranz et al. 2022). Notably, most previous studies do not account for variation in resource supply (but see O'Gorman et al. 2017), despite its central role in shaping food‐web size structure. Rather than indicating shortcomings of earlier studies, these comparisons underscore the challenges in extrapolating from spatially and temporally restricted results to broader taxonomic, spatial and temporal scales, and highlight the value of macroecological scales that jointly consider temperature and resource availability.

In Icelandic streams, O'Gorman et al. (2017) also found a higher relative abundance of large individuals in warmer streams, which they attributed to temperature‐driven increases in nutrient supply and basal resource carrying capacity that counteracted negative influences of temperature on body size (Yvon‐Durocher et al. 2011). An interesting difference in their study versus ours is that their positive temperature effect was detected with a univariate model. In our study, a univariate model with temperature revealed no relationship with λ (slope = 0.01 [−0.06 to 0.08]; 52% probability of a positive slope; Figure S11), despite a partial positive correlation between gross primary production and temperature (Figure 2). Instead, the positive temperature effect on λ occurred only through interactions with both gross primary production and organic matter. One explanation for this contrast is that, in Icelandic streams, there are minimal external organic matter inputs and in‐stream organic matter availability varies weakly with temperature and is instead structured primarily by disturbance and habitat chartacteristics (Junker et al. 2021). These factorslead to differences in both the covaration of resource supply with temperature and the nature of those resources (in‐stream production versus external organic matter inputs). In contrast, the broader spatial and environmental gradients in our study capture substantial covariation in OM availability, revealing that the positive temperature effect on λ in our study remains *after* accounting for resource supply. Together, these studies demonstrate that the response of food‐web size structure to temperature is entwined with resource supply across natural environmental gradients.

We hypothesize that resource supply modifies the metabolic constraints imposed by temperature, allowing food webs to support larger body sizes than expected under warming conditions. Changes in resource availability cause physiological adjustments that influence body size (McNab 2010), which in turn can alter λs. Evidence from stream ecosystems supports this mechanism: in a detritus‐based headwater stream, long‐term nutrient enrichment increased the abundance of large‐bodied macroinvertebrates while smaller taxa declined (Davis et al. 2010). This finding suggests that resource enrichment systematically favours larger consumers and contributes to the observed flattening of λ, particularly under conditions of elevated temperature where metabolic demands are high. Similarly, in stream ecosystems, Perkins et al. (2018) demonstrated that access to allochthonous terrestrial prey subsidies allowed top predators to escape within‐system energetic constraints. This access increased the relative abundance of larger‐bodied consumers and led to less negative λs than predicted by allometric scaling theory. These findings underscore how cross‐ecosystem energy inputs—particularly those disproportionately accessible to top consumers—can modify food‐web structure and generate deviations from expected size‐abundance scaling relationships under warming and nutrient‐enriched conditions.

Globally, stream and river ecosystems face increasing modifications to both thermal and resource regimes through changes in climate, land use and human water use (Johnson et al. 2024). These changes are expected to have widespread implications for the size structure and functioning of river ecosystems, though the extent of these changes will depend on how temperature and resources change interactively. For example, increasing temperatures are likely to coincide with increases in basal resource availability due to the strong relationship between GPP and temperature (Demars et al. 2011; Song et al. 2018; Figure 2b). Accordingly, it is increasingly clear that the effects of temperature on food‐web structure can be mediated by resource supply, particularly primary production, in some systems (Gjoni et al. 2023; Junker et al. 2020; O'Gorman et al. 2017). On the contrary, human alterations of riparian vegetation may have disproportionately strong and opposing effects, because removal or reduction in riparian plants can both increase stream temperature and reduce litter OM inputs (Webster et al. 1990). Based on our observations here, concurrent increases in temperature coupled with reductions in OM availability are likely to cause λ to become more negative, reducing the relative contribution of large individuals and the efficiency of energy transfer in food webs (Figure 3a, Figure S7). Our results therefore point to an important management implication: nature‐based solutions such as riparian buffer restoration may help mitigate the ecological impacts of warming by moderating stream temperature while sustaining resource subsidies that support food‐web size structure (Perkins et al. 2018).

Because our results are derived from streams spanning broad spatial and environmental gradients, they reflect macroecological patterns in which communities are adapted to local temperature and resource regimes. While warming in artificial aquatic systems is often associated with shifts toward smaller body sizes (Daufresne et al. 2009; Yvon‐Durocher et al. 2011), such findings may not directly translate to natural systems across broad spatial scales because organisms have adapted to those conditions. The main challenge moving forward is understanding the fundamental drivers that allow warmer food webs to support larger organisms, a pattern which, in this study, was robust to seasonality, taxonomic grouping and spatial autocorrelation. As climate change and anthropogenic pressures continue to alter resource availability and thermal regimes, understanding the interplay between trophic structure, species interactions and environmental change will be essential for predicting and mitigating long‐term ecosystem responses.

## Author Contributions

J.S.W., J.P.F.P. and J.R.J. designed the research. V.G., J.S.W., J.P.F.P. and J.R.J. performed the research. J.S.W. and J.R.F. analysed the data. V.G., J.S.W., J.P.F.P. and J.R.J. wrote the paper.

## Funding

This work was supported by the National Science Foundation (2106067).

## Supporting information


**Figure S1:** Overview of workflow to combine fish and macroinvertebrates. This is a visual summary of the steps taken to combine fish and macroinvertebrate data sets of individual body sizes. The results of these steps formed the data used in analyses of individual size distributions (i.e., size spectra).
**Figure S2:** Example of resampling body sizes. To facilitate plotting and model checking, we resampled data with replacement, weighted by the density of sizes. In this simplified example, there are two size densities (1.8 and 0.2). After resampling, the body sizes associated with size density 1.8 are represented more often than the body sizes with size density 0.2, and all sizes now have the same implied density. This ensured that the body masses were represented in proportion to their density in the stream prior to model checking (e.g., Figure S4) and prior to plotting ISD's (e.g., Figure S6).
**Figure S3:** Three‐hundred simulations from the prior predictive distribution. Lines show possible relationships between λ and temperature with GPP and organic matter set to their median, low (median—1 SD) and high (median + 1 SD) values. The priors largely limit λ to values between ~−3 and −1 but allow for a wide range of possible relationships with mean annual temperature. mat_s is mean annual temperature (standardized as a z‐score).
**Figure S4:** Posterior predictive model checking. (a) Densities of 10 simulated data sets (light blue) compared to the raw data (dark blue). The strong overlap indicates that the model has good fit because it can generate body size distributions that mimic the raw data. Before plotting, the raw data were resampled with replacement 10,000 times and weighted to density to account for the different collection techniques and implied densities among macroinvertebrates and fish. (b) Same as (a), but with samples produced for each stream site.
**Figure S5:** Posterior predictive checks and prior sensitivity. (a) Geometric mean body sizes from the raw data compared to the posterior predictive distributions for all 159 samples. Raw data have a single value per sample (black dot). Posterior predictions (*y*_rep_) show the median ±95% credible intervals. (b) Parameter values from the same model with either informative priors (as reported in the main text) or with default priors in *brms* that are less informative. Results show almost no influence of the informative priors on the analysis.
**Figure S6:** Fitted versus observed individual size distributions. Twenty‐one randomly chosen samples illustrating model fit to data. Dots are individual body masses. Red lines show the posterior median and 95% CrI (barely visible). Panel labels indicate the NEON site and sample number. Dots have been resampled to correct for variation in density (no/m^2^) between fish and macroinvertebrates. In most cases, this process excludes the largest individuals in the plot, because they are rare. Hence the range of dry masses is likely wider in the raw data compared to the re‐sampled data.
**Figure S7:** Conditional slopes of λ with mean annual stream temperature across different levels of resource supply: annual gross primary production (GPP) and organic matter (OM). Slopes become more positive as OM increases (comparing a row across columns), but not as GPP increases (comparing a column across rows). This suggests that OM is a primary factor influencing the change in λ across temperature. Values of ‘Low’, ‘Median’ and ‘High’ represent the 25th, 50th and 75th percentiles of OM or GPP.
**Figure S8:** Consistency in the positive relationship between mean annual stream temperatures and the ISD exponent λ. Posterior median slopes (95% CrI) are shown for each year, holding organic matter and gross primary production at their median values. The probability that slopes are positive ranges from 84% in 2021 to > 96% in 2017, 2019, 2020.
**Figure S9:** Consistency in the interactive effects of mean annual stream temperatures, mean annual gross primary production (GPP) and standing stock organic matter (OM) on the ISD exponent λ. Posterior medians (95% CrI) are shown for each year, temperature and resource combination. Values of ‘Low’, ‘Median’ and ‘High’ represent the 25th, 50th and 75th percentiles of OM or GPP.
**Figure S10:** Comparing seasonal, taxonomic and spatial effects. To determine how seasonal, taxonomic, methodological and spatial effects affected the analysis we re‐ran the model in the main text five times: once on only the first samples from each year (‘First samples only’), once on the last samples from each year (‘Last samples only’), once with only macroinvertebrates (‘Macroinvertebrates’), once with only fish (‘Fish only’), once with only a single sampler type (‘Surber’) for macroinvertebrates, and once with a Gaussian process varying intercept to account for spatial autocorrelation (‘Spatial’). In each column, the results from the main text are shown in black. The coloured shading with white lines show the 95% CrI and median slope of the six re‐fit models.
**Figure S11:** Regression from a univariate model with temperature as the only fixed predictor. This model implies no relationship between λ and mean annual stream temperature, presumably because the model does not account for variation in resource supply, as in Figure S7. The small dots are posterior median λs from individual samples. The larger dots and error bars are site specific medians and 95% CrI.
**Table S1:** List of NEON data product used in this work. See Supplementary Information S1 for the bibliography of the sources. Macroinvertebrates and fish were used to obtain body sizes and densities. Temperature, stream discharge and oxygen were used to estimate Gross Primary Production. Temperature was also used to estimate mean annual temperature. Organic matter was measured directly using samples from the NEON Biorepository.

## Data Availability

Data and code are available at https://zenodo.org/records/20704567.
