# Supplementary material for: Temperature and Resource Supply Drive Continental Variation in Size Structure of Freshwater Food Webs
Source: Ecol Lett. 2026 Jun 24;29(6):e70437. doi: 10.1111/ele.70437 (PMC13291765; doi:10.1111/ele.70437)
Supplement: Supplementary file 1 — Figure S1: Overview of workflow to combine fish and macroinvertebrates. This is a visual summary of the steps taken to combine fish and macroinvertebrate data sets of individual body sizes. The results of these steps formed the data used in analyses of individual size distributions (i.e., size spectra). Figure S2: Example of resampling body sizes. To facilitate plotting and model checking, we resampled data with replacement, weighted by the density of sizes. In this simplified example, there are two size densities (1.8 and 0.2). After resampling, the body sizes associated with size density 1.8 are represented more often than the body sizes with size density 0.2, and all sizes now have the same implied density. This ensured that the body masses were represented in proportion to their density in the stream prior to model checking (e.g., Figure S4) and prior to plotting ISD's (e.g., Figure S6). Figure S3: Three‐hundred simulations from the prior predictive distribution. Lines show possible relationships between λ and temperature with GPP and organic matter set to their median, low (median—1 SD) and high (median + 1 SD) values. The priors largely limit λ to values between ~−3 and −1 but allow for a wide range of possible relationships with mean annual temperature. mat_s is mean annual temperature (standardized as a z‐score). Figure S4: Posterior predictive model checking. (a) Densities of 10 simulated data sets (light blue) compared to the raw data (dark blue). The strong overlap indicates that the model has good fit because it can generate body size distributions that mimic the raw data. Before plotting, the raw data were resampled with replacement 10,000 times and weighted to density to account for the different collection techniques and implied densities among macroinvertebrates and fish. (b) Same as (a), but with samples produced for each stream site. Figure S5: Posterior predictive checks and prior sensitivity. (a) Geometric mean body sizes from the raw data compar [file ELE-29-0-s001.docx]

**Supporting Information for**

Temperature and resource supply drive continental variation in size structure of freshwater food webs

**Vojsava Gjoni^1,2^, Justin P. F. Pomeranz^3^, James R. Junker^4^, Jeff S. Wesner^1^**

*^1^University of South Dakota, Department of Biology, Vermillion, SD, USA 57069*

*^2^Institute of Marine Biological Resources and Biotechnology of the National Research Council, Mazara del Vallo, Italy*

*^3^Colorado Mesa University, Department of Physical and Environmental Sciences, Grand Junction, CO, USA 81501*

*^4^University of North Texas, Department of Biological Sciences, Denton, TX, USA 76203*

**Corresponding e-mail: vojsavagjoni@cnr.it*

**This PDF file includes:**

Supporting text

Figures S1 to S8

Tables S1 to S2

SI References

**Other supporting materials for this manuscript include the following:**

All data and code are available here: <https://zenodo.org/records/20704567>

Supporting Information Text

## Model Checking

We examined model fit using Bayesian R-squared(Gelman *et al.* 2019), posterior predictive checks(Gabry *et al.* 2019), and Bayesian P-values(Hooten & Hobbs 2015). Each of these measures summarize comparisons between the raw data and data predicted from the posterior distribution of the fitted model. To do this, we first needed to remove the $counts$ variable from the raw data. It contains the density of each individual body size, which allows us to combine the fish and macroinvertebrate data sets while accounting for the different collection areas and relative abundances of each taxa(Wesner & Pomeranz 2023b). However, while the densities are included in the likelihood when fitting the model, they are not included in the random number generator for simulating data. Therefore, to remove them, we re-sampled 5,000 individual body sizes with replacement from each of the 159 samples, weighting each sample by its density in no/m^2^. This generated a vector of individual body sizes, each with an implied density of 1.

To simulate new data from the posterior, we first extracted the posterior distribution of $\lambda$ for each of the $j=$ 159 samples using the add_epred_draws() function from the *tidybayes* package (Kay 2023). This function applies the following:

$$\lambda_{j}^{k}=g\left( \boldsymbol{\theta}_{j}^{k},\text{X}_{j} \right)$$

where $\lambda_{j}^{k}$ is the $k^{\text{th}}$ posterior draw from sample $j$, derived from the linear equation containing the $k^{\text{th}}$ parameter values $\boldsymbol{\theta}$ and data $\text{X}$ associated with sample $j$. From the first 100 $k$ draws of each $\lambda_{j}$, we simulated 5,000 individual body sizes using the inverse cumulative density function(Wesner *et al.* n.d.) via the rparetocounts() function from the R package *isdbayes*(Wesner & Pomeranz 2023b).

The result is 5000 simulated individual body sizes from each of the 159 NEON samples. This allowed us to compare model fit at the sample level. We also compared fits from the full model using the posterior mean estimate of $\lambda$. In other words, we simulated the full data set rather than data sets for each sample $j$.

To determine how well the model recaptures the raw data, we visually compared the simulated data, $y_{pred,j}$, and the raw re-sampled data, $y_{j}$ of each $j$ sample. We also calculated the geometric mean for each $j$ prediction and raw data. We then calculated a Bayesian P-value as the proportion of posterior draws for which the geometric mean was greater than the raw value. Proportions <.1 or >0.9 are generally indicative of poor model fit, indicating a mismatch in the $y_{pred}$ and $y$(Hooten & Hobbs 2015).

We calculated a Bayesian R^2^ using the following formula(Gelman *et al.* 2019):

$$R^{2}=\frac{\text{V}_{ypred}}{\text{V}_{ypred}+\text{V}_{res}},$$

where $\text{V}_{ypred}$ is the variance of $y_{pred}$ and $\text{V}_{res}$ is the variance of the residuals $y_{pred}-y$. We repeated this equation for each of 1,000 $k$ draws from the posterior, generating a distribution of $R^{2}$.

Finally, to visualize the size spectrum, we plotted the cumulative distribution function from the fitted model against the raw data(Edwards, Andrew 2023). In particular, we obtained the posterior median and 95% CrI of $\lambda$ for each of the 159 body size samples. From those, we calculated the following cumulative distribution function(Edwards *et al.* 2017b):

$$\text{P}\left( X\geq x \right)=\frac{1-\left( x^{\lambda+1}-\left( \text{xmin}^{\lambda+1} \right) \right)}{\left( \text{xmax}^{\lambda+1}-\text{xmin}^{\lambda+1} \right)}$$

where $\text{P}\left( X\geq x \right)$ is the probability of obtaining a body size $X$ that is greater than or equal to a give size $x$ in the data set. The largest individual in the data set has $\text{P}\left( X\geq x \right)=1$. The smallest individual has $\text{P}\left( X\geq x \right)=0$, and all other individuals are in between. To plot the raw data on top of this function, we ranked body sizes within samples from 1 to 5000 (largest to smallest). We then multiplied $\text{P}\left( X\geq x \right)$ by 5000 so that the y-axis contains the number of individuals $\geq x$, rather that a probability *per se*. Because the raw re-sampled data are unlikely to contain the true $x_{max}$ or $x_{min}$ we used the $x_{max}$ and $x_{min}$of the re-sampled data in the calculation for the cumulative distribution function.

*Results*

The model had a Bayesian R^2^ of 0.32 $\pm$ 0.06 (mean $\pm$ sd), explaining ~32% of the variance of new data. Posterior predictive checks revealed generally good fit as the predictive distributions closely resemble the raw re-sampled distributions (Figure S6). This suggests that the truncated Pareto is a reasonable likelihood for these data. There is also strong agreement in the geometric means (GM) across samples. Figure S6 shows the GM($y_{pred}$) compared to the GM($y$) across all 159 samples. The GM($y$) is consistently within the 95% credible intervals of GM($y_{pred}$) at each sample (Figure S6). Moreover, Bayesian P-values across all samples ranged from 0.02 to 1 with a mean of 0.5 and sd of 0.3, again indicating good overall model fit.

*Data Sources*

We used six data sources collected by the National Ecological Data Observatory (Table S2): Macroinvertebrates(NEON 2023c), Fish(NEON 2022), Temperature(NEON 2023d), Stream Discharge(NEON 2023a), Oxygen(NEON 2023b), and Organic Matter, which was measure directly from samples maintained at the NEON Biorepository(Yule *et al.* 2020).


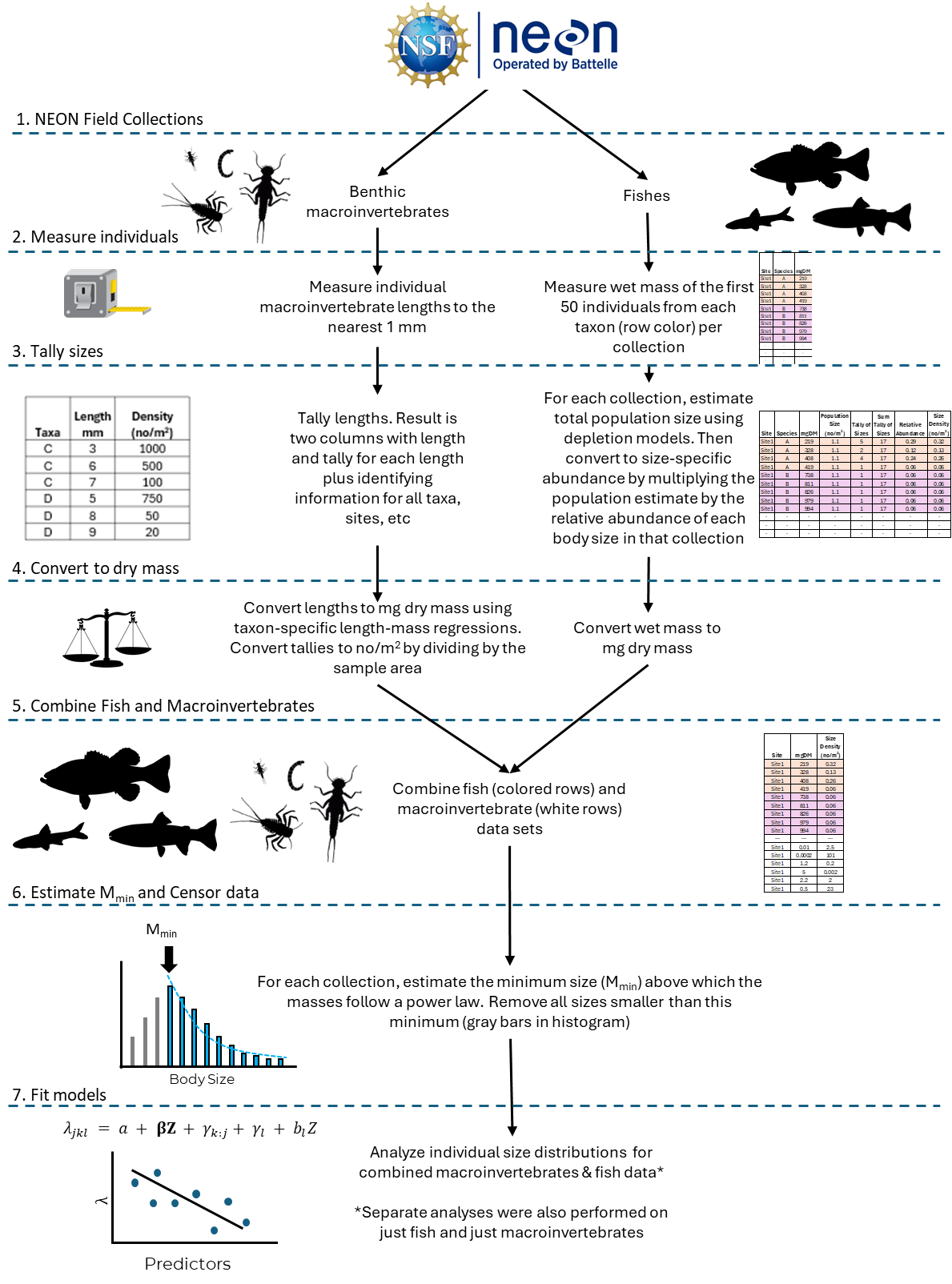


**Fig. S1. Overview of workflow to combine fish and macroinvertebrates**. This is a visual summary of the steps taken to combine fish and macroinvertebrate data sets of individual body sizes. The results of these steps formed the data used in analyses of individual size distributions (i.e., size spectra).


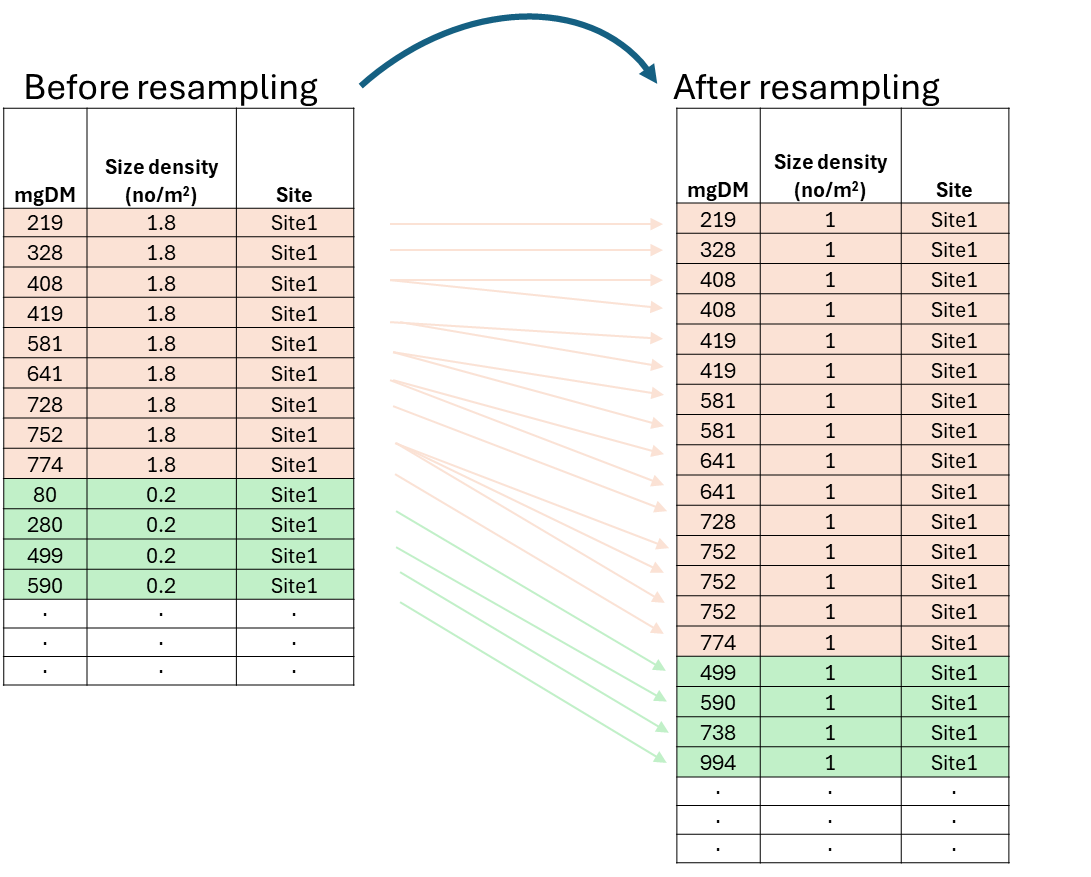


**Fig. S2. Example of resampling body sizes.** To facilitate plotting and model checking, we resampled data with replacement, weighted by the density of sizes. In this simplified example, there are two size densities (1.8 and 0.2). After resampling, the body sizes associated with size density 1.8 are represented more often than the body sizes with size density 0.2, and all sizes now have the same implied density. This ensured that the body masses were represented in proportion to their density in the stream prior to model checking (e.g., Fig. S4) and prior to plotting ISD’s (e.g., Fig. S6).


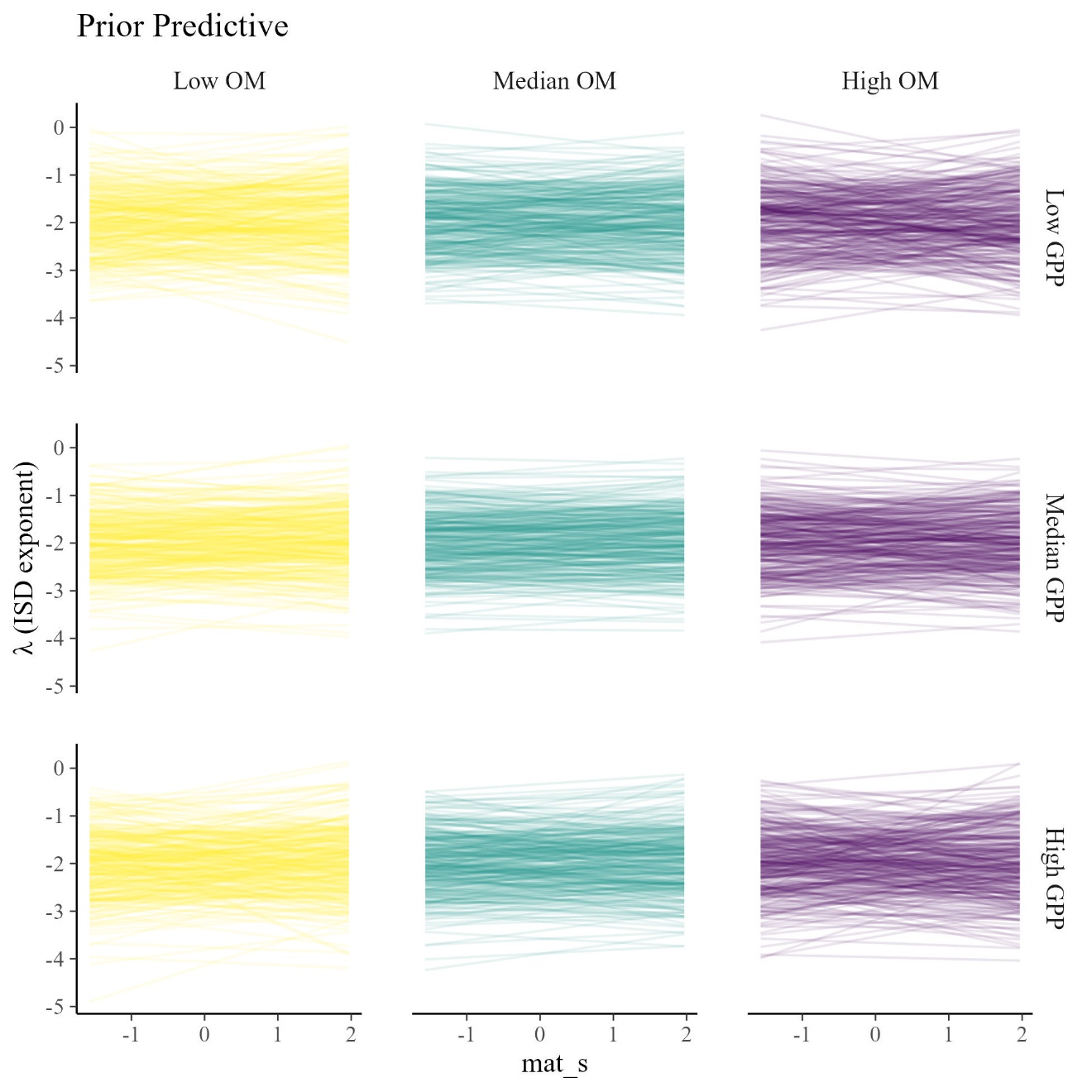


**Fig. S3. Three-hundred simulations from the prior predictive distribution.** Lines show possible relationships between λ and temperature with GPP and organic matter set to their median, low (median - 1sd) and high (median + 1sd) values. The priors largely limit λ to values between ~-3 to -1 but allow for a wide range of possible relationships with mean annual temperature. mat_s is mean annual temperature (standardized as a z-score).


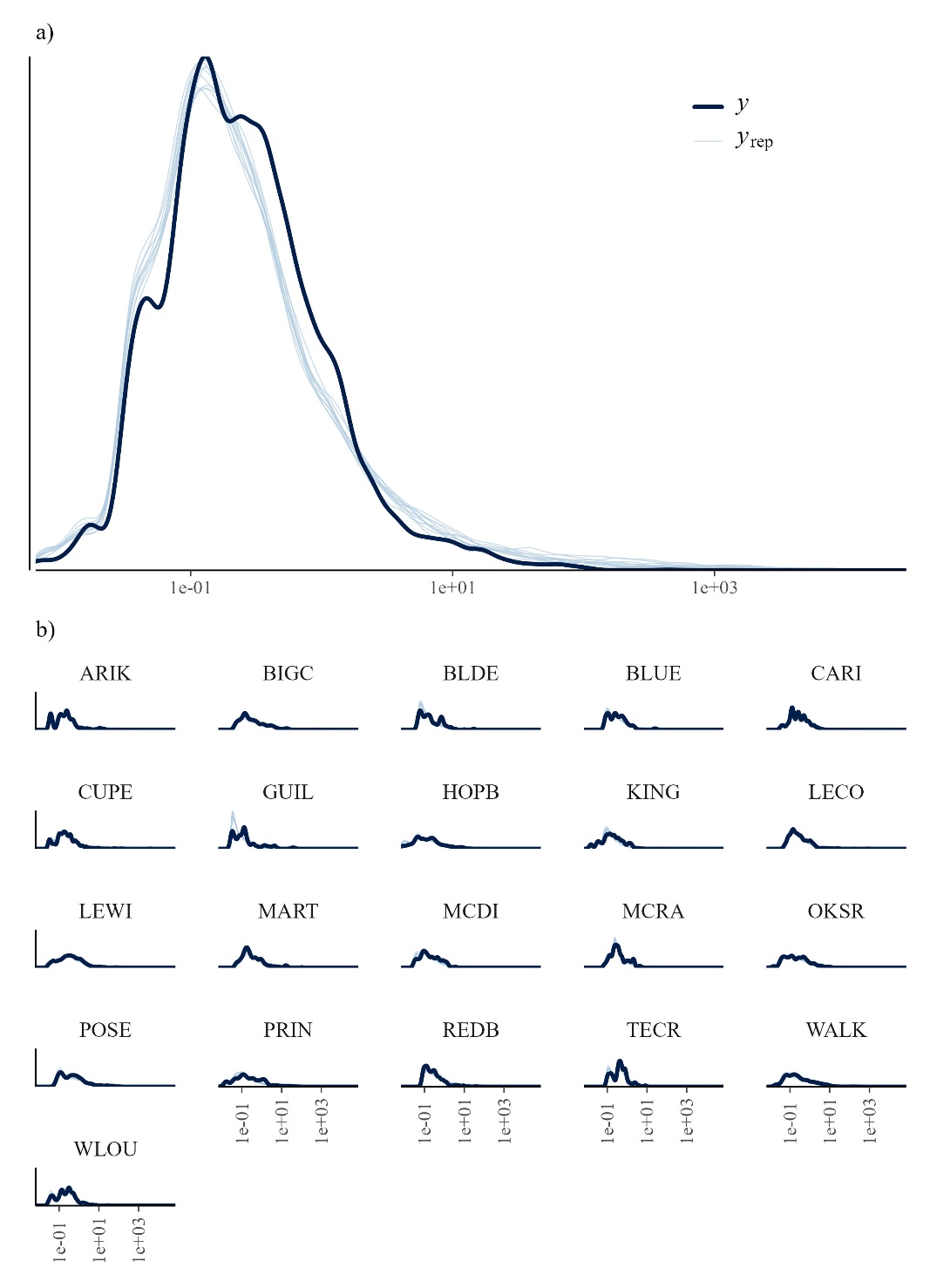


**Fig. S4. Posterior predictive model checking.** a) Densities of ten simulated data sets (light blue) compared to the raw data (dark blue). The strong overlap indicates that the model has good fit because it can generate body size distributions that mimic the raw data. Before plotting, the raw data were resampled with replacement 10,000 times and weighted to density to account for the different collection techniques and implied densities among macroinvertebrates and fish. b) Same as a), but with samples produced for each stream site.


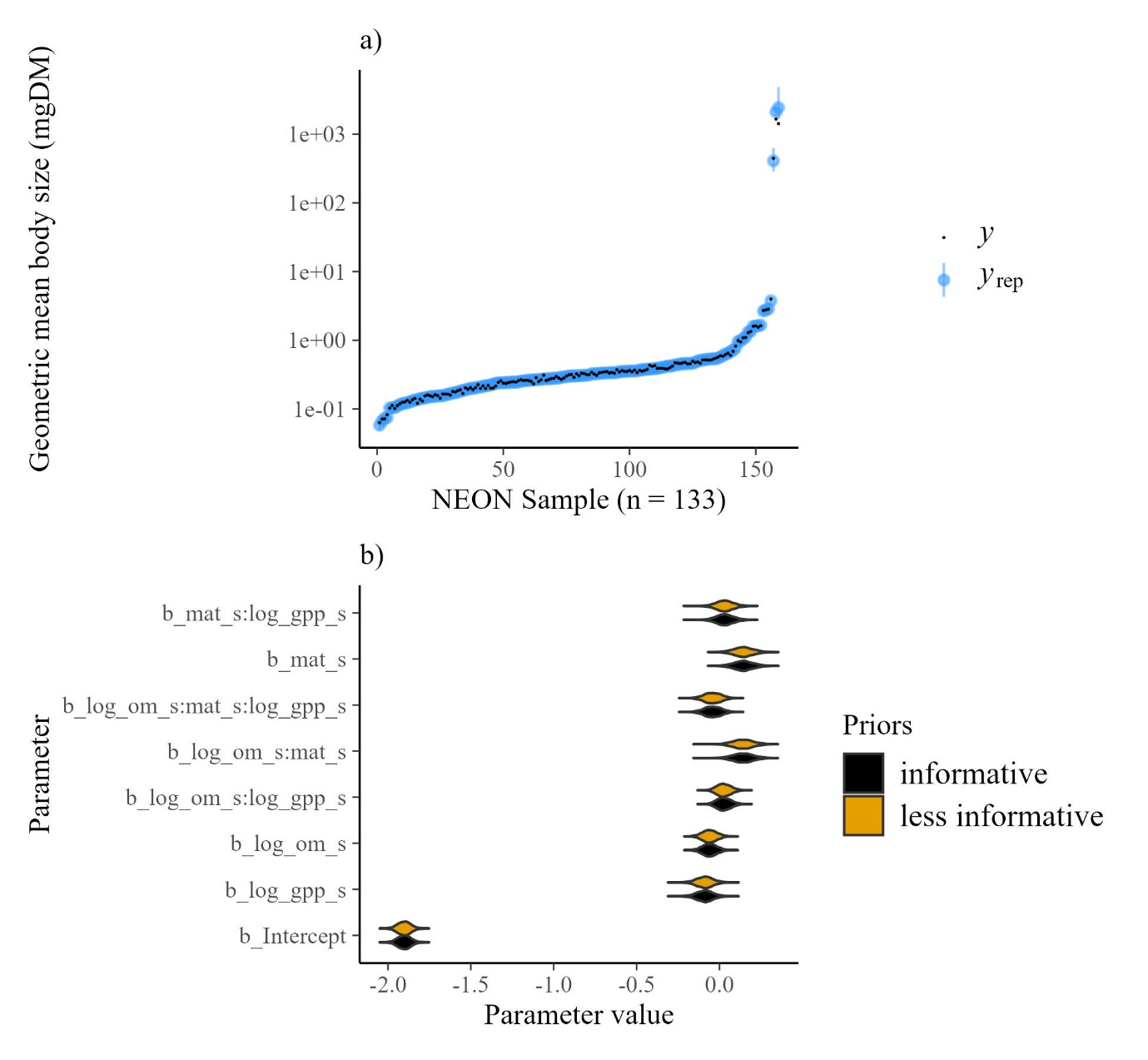


**Fig. S5. Posterior predictive checks and prior sensitivity.** a) Geometric mean body sizes from the raw data compared to the posterior predictive distributions for all 159 samples. Raw data have a single value per sample (black dot). Posterior predictions (*y*_rep_) show the median ± 95% credible intervals. b) Parameter values from the same model with either informative priors (as reported in the main text) or with default priors in *brms* that are less informative. Results show almost no influence of the informative priors on the analysis.


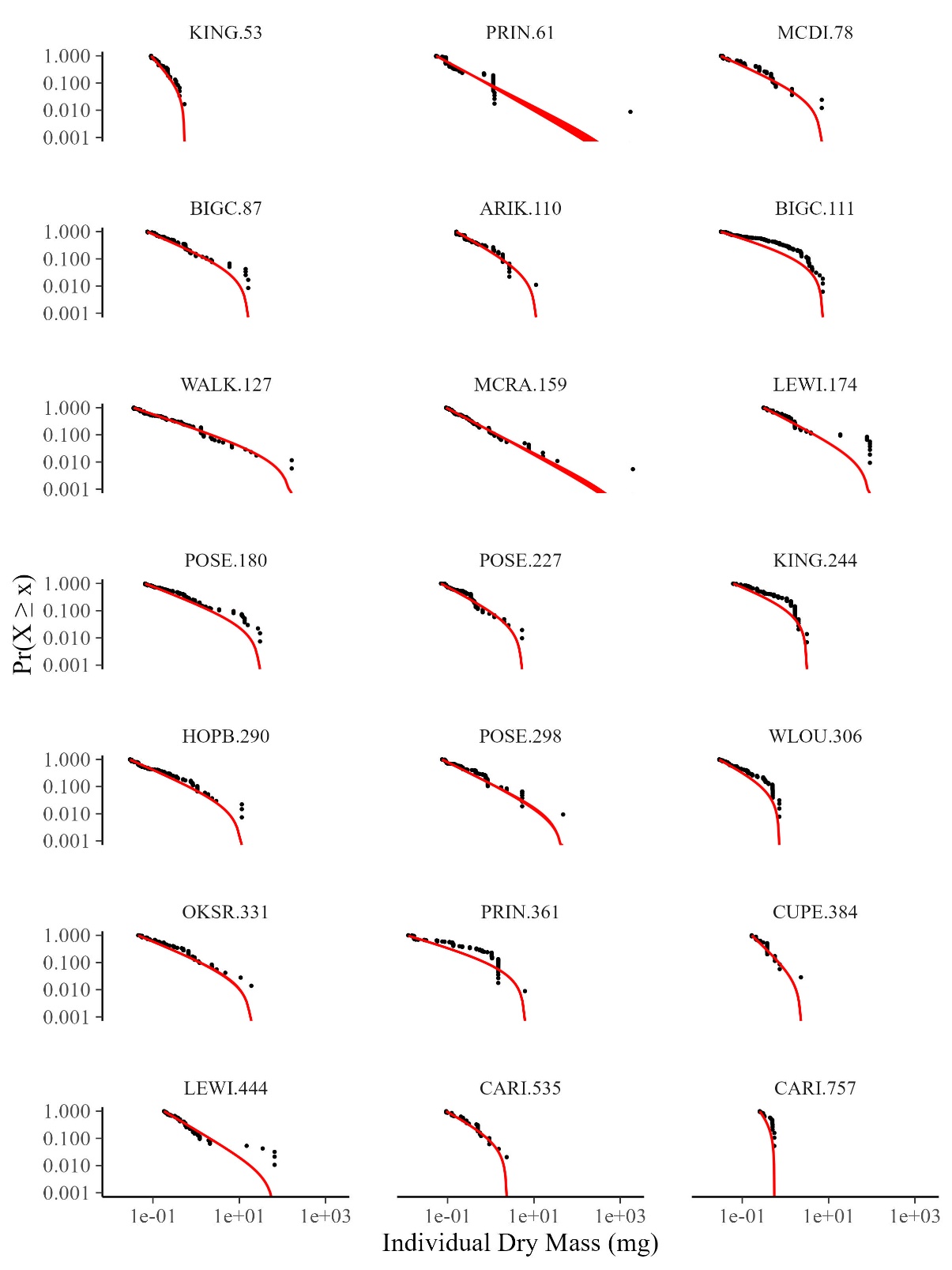


**Fig. S6. Fitted versus observed individual size distributions.** Twenty-one randomly chosen samples illustrating model fit to data. Dots are individual body masses. Red lines show the posterior median and 95% CrI (barely visible). Panel labels indicate the NEON site and sample number. Dots have been resampled to correct for variation in density (no/m^2^) between fish and macroinvertebrates. In most cases, this process excludes the largest individuals in the plot, because they are rare. Hence the range of dry masses is likely wider in the raw data compared to the re-sampled data.

**
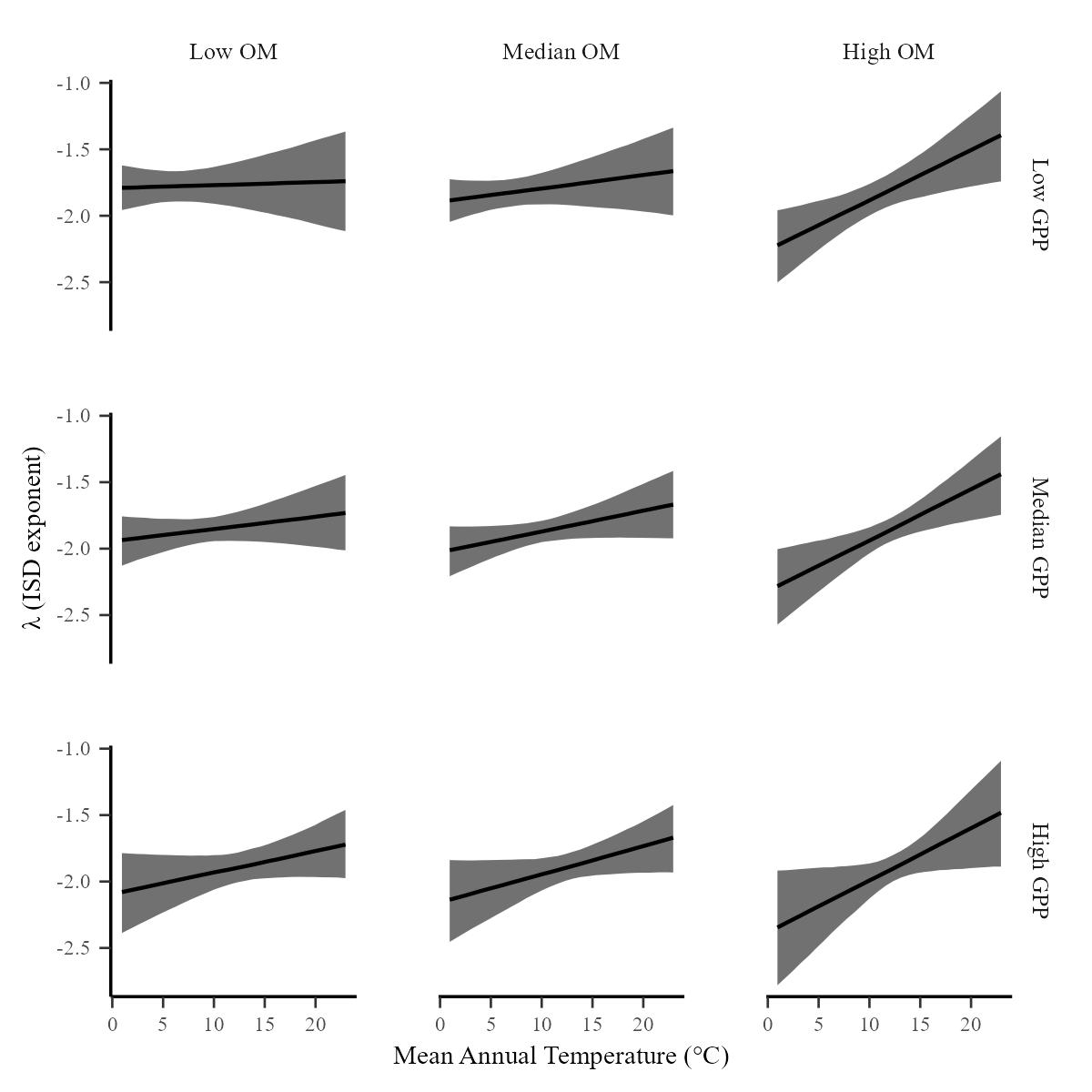
**

**Fig. S7. Conditional slopes of λ with mean annual stream temperature across different levels of resource supply: annual gross primary production (GPP) and organic matter (OM)**. Slopes become more positive as OM increases (comparing a row across columns), but not as GPP increases (comparing a column across rows). This suggests that OM is a primary factor influencing the change in λ across temperature. Values of “Low”, “Median”, and “High” represent the 25^th^, 50^th^, and 75^th^ percentiles of OM or GPP.


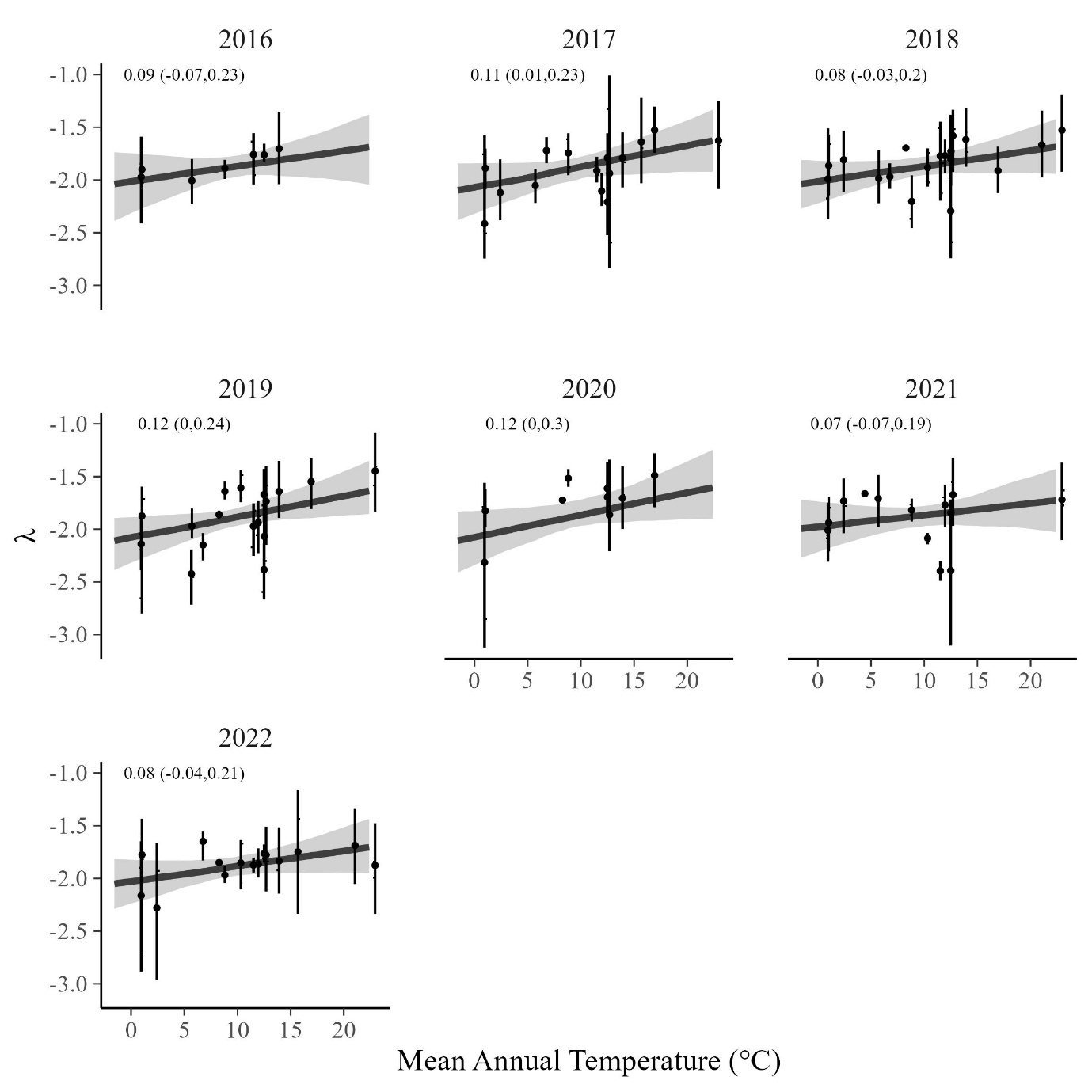


**Fig. S8.** Consistency in the positive relationship between mean annual stream temperatures and the ISD exponent λ. Posterior median slopes (95% CrI) are shown for each year, holding organic matter and gross primary production at their median values. The probability that slopes are positive ranges from 84% in 2021 to >96% in 2017, 2019, 2020.


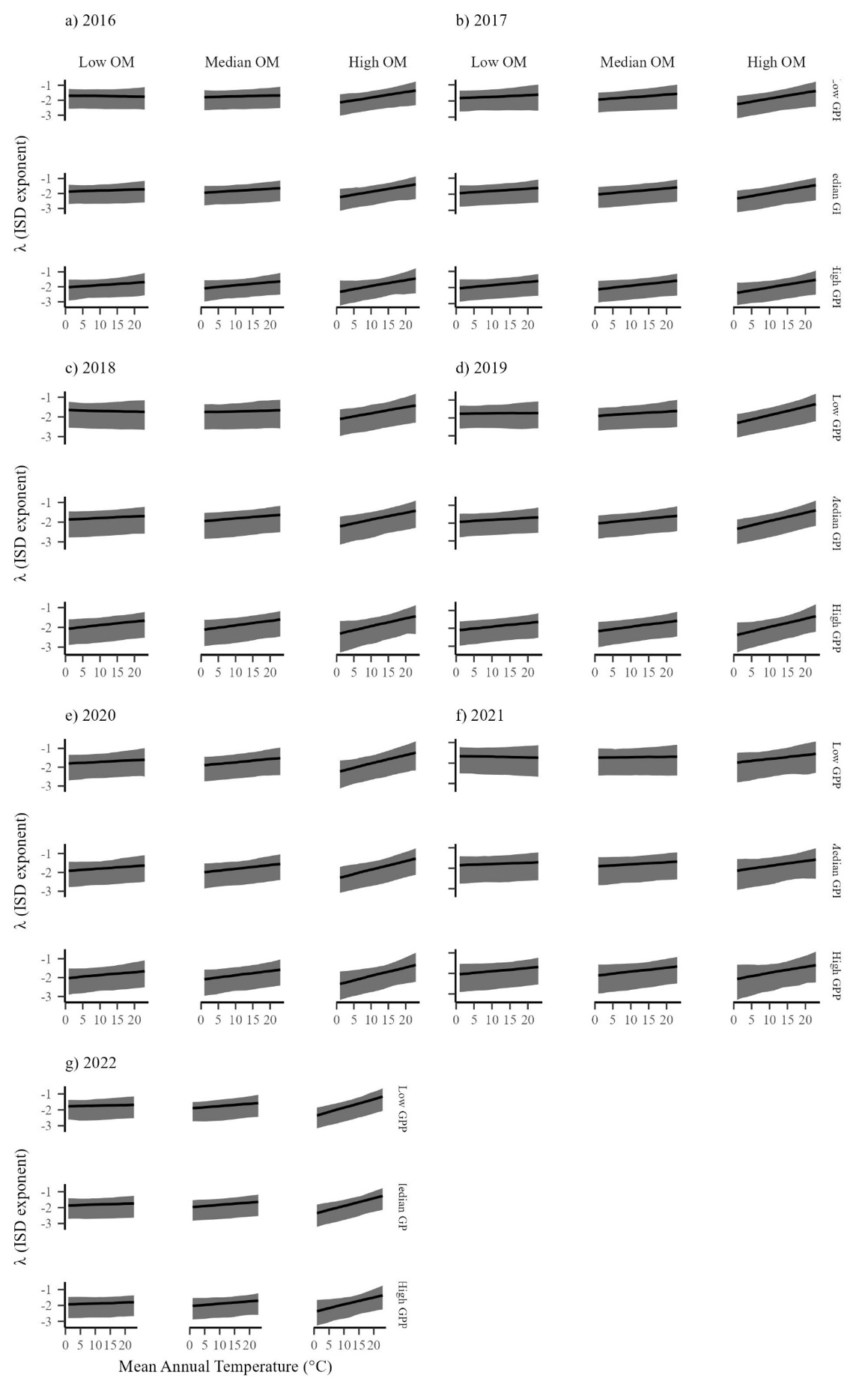


**Fig. S9.** Consistency in the interactive effects of mean annual stream temperatures, mean annual gross primary production (GPP), and standing stock organic matter (OM) on the ISD exponent λ. Posterior medians (95% CrI) are shown for each year, temperature, and resource combination. Values of “Low”, “Median”, and “High” represent the 25^th^, 50^th^, and 75^th^ percentiles of OM or GPP.


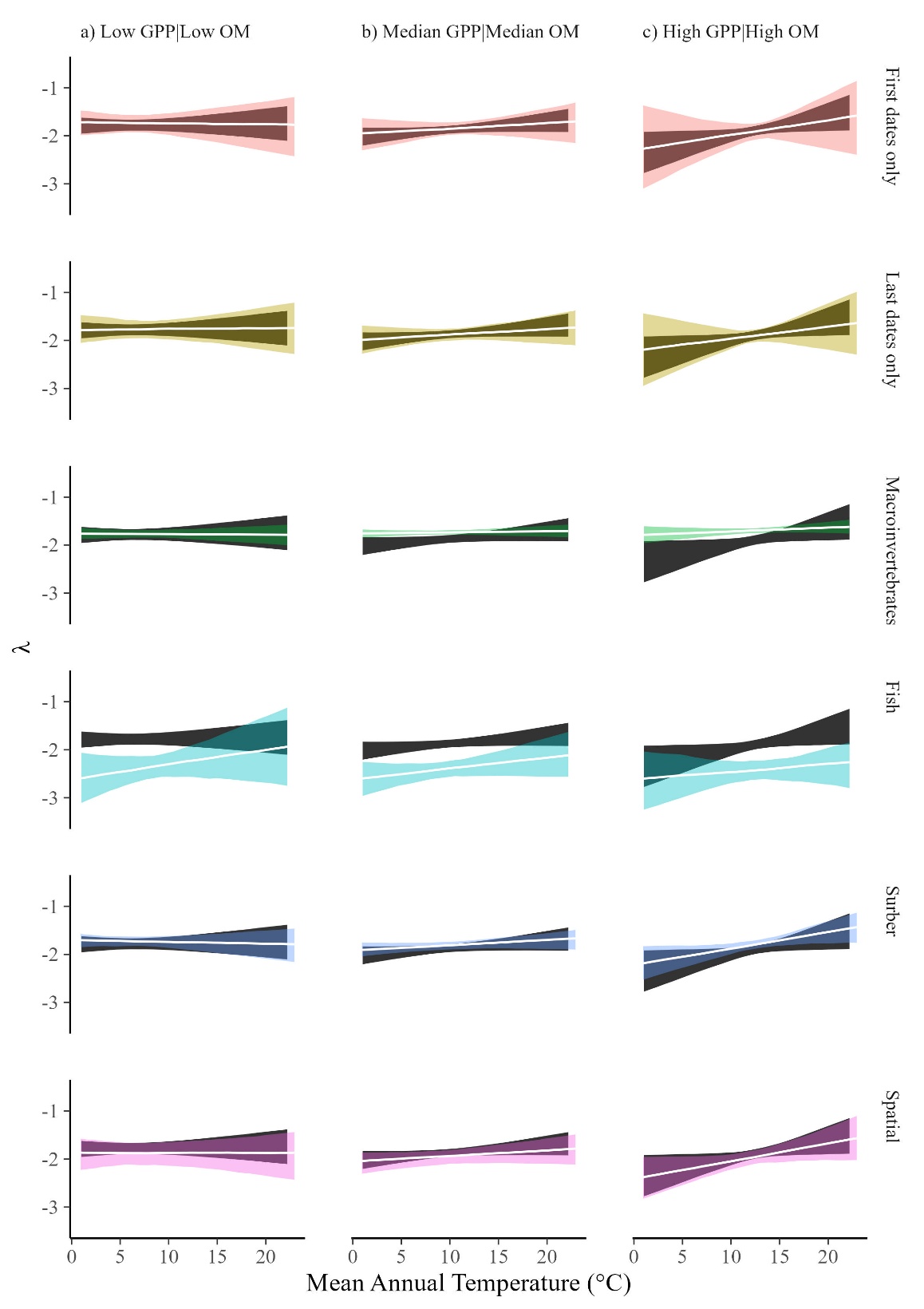


**Fig. S10. Comparing seasonal, taxonomic, and spatial effects.** To determine how seasonal, taxonomic, methodological, and spatial effects affected the analysis we re-ran the model in the main text five times: once on only the first samples from each year (“First samples only”), once on the last samples from each year (“Last samples only”), once with only macroinvertebrates (“Macroinvertebrates”), once with only fish (“Fish only”), once with only a single sampler type (“Surber”) for macroinvertebrates, and once with a Gaussian process varying intercept to account for spatial autocorrelation (“Spatial”). In each column, the results from the main text are shown in black. The colored shading with white lines show the 95% CrI and median slope of the six re-fit models.

**
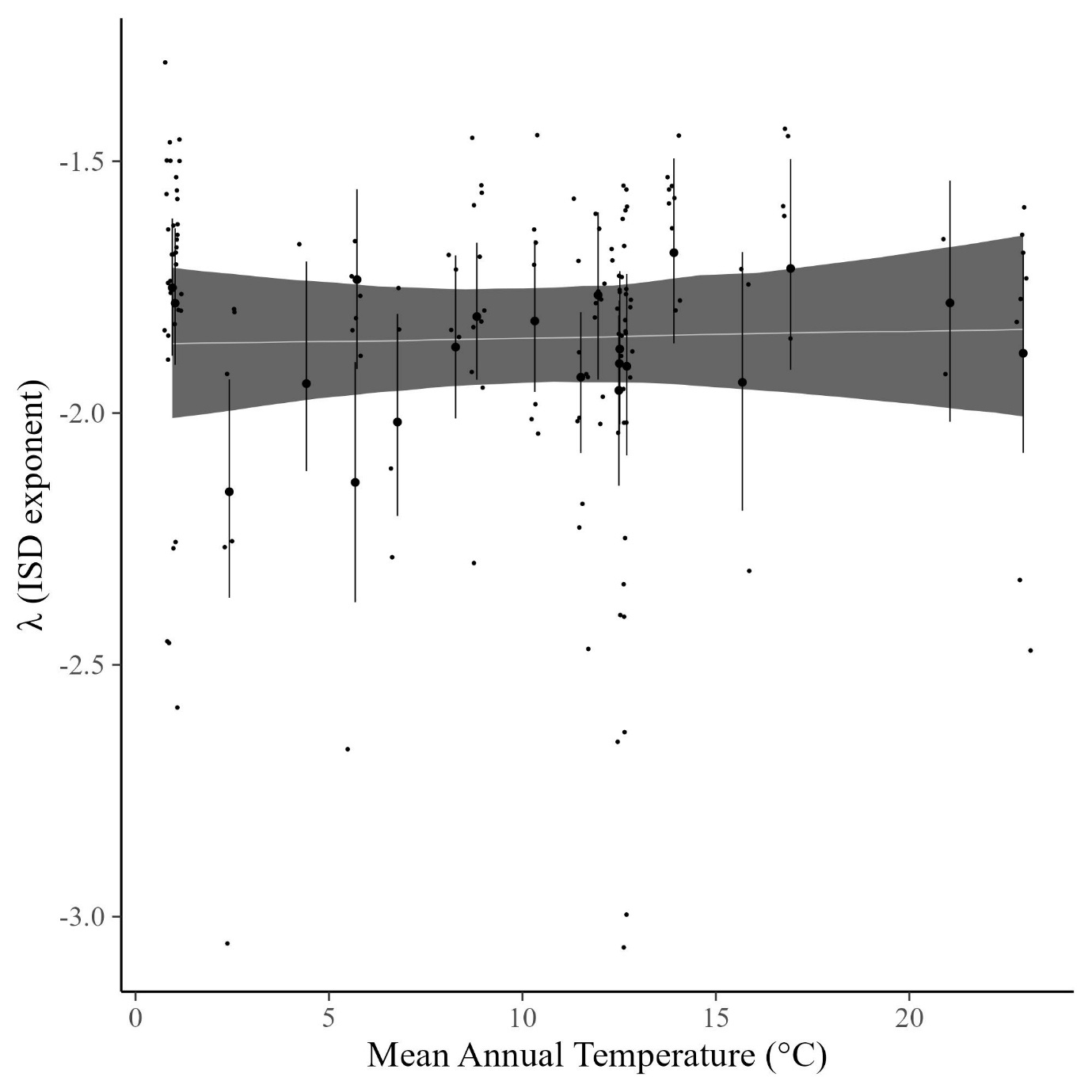
**

**Fig. S11. Regression from a univariate model with temperature as the only fixed predictor**. This model implies no relationship between λ and mean annual stream temperature, presumably because the model does not account for variation in resource supply, as in Figure S7. The small dots are posterior median λs from individual samples. The larger dots and error bars are site specific medians and 95% CrI.

| Table S1. List of NEON data product used in this work. See Supplementary Information for the bibliography of the sources. Macroinvertebrates and fish were used to obtain body sizes and densities. Temperature, stream discharge and oxygen were used to estimate Gross Primary Production. Temperature was also used to estimate mean annual temperature. Organic matter was measured directly using samples from the NEON Biorepository. | | | |
| --- | --- | --- | --- |
| NEON ID | Description | Link | Source |
| DP1.20120.001 | Macroinvertebrates | <https://data.neonscience.org/data-products/DP1.20120.001> | ^7^ |
| DP1.20107.001 | Fish | <https://data.neonscience.org/data-products/DP1.20107.001> | ^8^ |
| DP1.20053.001 | Temperature | <https://data.neonscience.org/data-products/DP1.20053.001> | ^9^ |
| DP4.00130.001 | Stream Discharge | <https://data.neonscience.org/data-products/DP4.00130.001> | ^10^ |
| DP1.20288.001 | Oxygen | <https://data.neonscience.org/data-products/DP1.20288.001> | ^11^ |
| Biorepository | Organic matter | https://biorepo.neonscience.org/%20portal/misc/cite.php | ^12^ |

**SI References**

Appling, A.P., Hall Jr., R.O., Yackulic, C.B. & Arroita, M. (2018). Overcoming Equifinality: Leveraging Long Time Series for Stream Metabolism Estimation. *J. Geophys. Res. Biogeosciences*, 123, 624–645.

Arranz, I., Grenouillet, G. & Cucherousset, J. (2023a). Biological invasions and eutrophication reshape the spatial patterns of stream fish size spectra in France. *Divers. Distrib.*, 29, 590–597.

Arranz, I., Grenouillet, G. & Cucherousset, J. (2023b). Human pressures modulate climate-warming-induced changes in size spectra of stream fish communities. *Nat. Ecol. Evol.*, 7, 1072–1078.

Bar-On, Y.M., Phillips, R. & Milo, R. (2018). The biomass distribution on Earth. *Proc. Natl. Acad. Sci.*, 115, 6506–6511.

Blanchard, J.L., Heneghan, R.F., Everett, J.D., Trebilco, R. & Richardson, A.J. (2017). From Bacteria to Whales: Using Functional Size Spectra to Model Marine Ecosystems. *Trends Ecol. Evol.*, 32, 174–186.

Brose, U., Blanchard, J.L., Eklöf, A., Galiana, N., Hartvig, M., R. Hirt, M., *et al.* (2017). Predicting the consequences of species loss using size-structured biodiversity approaches. *Biol. Rev.*, 92, 684–697.

Brown, J.H., Gillooly, J.F., Allen, A.P., Savage, V.M. & West, G.B. (2004). Toward a Metabolic Theory of Ecology. *Ecology*, 85, 1771–1789.

Bürkner, P.-C. (2017). brms: An R Package for Bayesian Multilevel Models Using Stan. *J. Stat. Softw.*, 80, 1–28.

Cross, W.F., Hood, J.M., Benstead, J.P., Huryn, A.D. & Nelson, D. (2015). Interactions between temperature and nutrients across levels of ecological organization. *Glob. Change Biol.*, 21, 1025–1040.

Cyr, H. & Pace, M.L. (1993). Allometric Theory: Extrapolations from Individuals to Communities. *Ecology*, 74, 1234–1245.

Daufresne, M., Lengfellner, K. & Sommer, U. (2009). Global warming benefits the small in aquatic ecosystems. *Proc. Natl. Acad. Sci. U. S. A.*, 106, 12788–12793.

Davis, J.M., Rosemond, A.D., Eggert, S.L., Cross, W.F. & Wallace, J.B. (2010). Nutrient enrichment differentially affects body sizes of primary consumers and predators in a detritus-based stream. *Limnol. Oceanogr.*, 55, 2305–2316.

Demars, B.O., Russell Manson, J., Ólafsson, J.S., Gíslason, G.M., Gudmundsdóttir, R., Woodward, G., *et al.* (2011). Temperature and the metabolic balance of streams. *Freshw. Biol.*, 56, 1106–1121.

Dickie, L.M., Kerr, S.R. & Boudreau, P.R. (1987). Size-Dependent Processes Underlying Regularities in Ecosystem Structure. *Ecol. Monogr.*, 57, 233–250.

Dossena, M., Yvon-Durocher, G., Grey, J., Montoya, J.M., Perkins, D.M., Trimmer, M., *et al.* (2012). Warming alters community size structure and ecosystem functioning. *Proc. R. Soc. B Biol. Sci.*, 279, 3011–3019.

Edwards, A.M., Robinson, J.P.W., Plank, M.J., Baum, J.K. & Blanchard, J.L. (2017a). Testing and recommending methods for fitting size spectra to data. *Methods Ecol. Evol.*, 8, 57–67.

Edwards, A.M., Robinson, J.P.W., Plank, M.J., Baum, J.K. & Blanchard, J.L. (2017b). Testing and Recommending Methods for Fitting Size Spectra to Data. *Methods Ecol. Evol.*, 8, 57–67.

Edwards, Andrew. (2023). sizeSpectra: Fitting Size Spectra to Ecological Data Using Maximum Likelihood.

Elhacham, E., Ben-Uri, L., Grozovski, J., Bar-On, Y.M. & Milo, R. (2020). Global human-made mass exceeds all living biomass. *Nature*, 588, 442–444.

Enquist, B.J., Erwin, D., Savage, V. & Marquet, P.A. (2024). Scaling approaches and macroecology provide a foundation for assessing ecological resilience in the Anthropocene. *Philos. Trans. R. Soc. B Biol. Sci.*, 379, 20230010.

Gabry, J., Simpson, D., Vehtari, A., Betancourt, M. & Gelman, A. (2019). Visualization in Bayesian Workflow. *J. R. Stat. Soc. Ser. A Stat. Soc.*, 182, 389–402.

Gelman, A., Goodrich, B., Gabry, J. & Vehtari, A. (2019). R-squared for Bayesian Regression Models. *Am. Stat.*, 73, 307–309.

Gillespie, C. (2024). Package ‘poweRlaw.’

Hatton, I.A., Heneghan, R.F., Bar-On, Y.M. & Galbraith, E.D. (2021). The global ocean size spectrum from bacteria to whales. *Sci. Adv.*, 7, eabh3732.

Hooten, M.B. & Hobbs, N.T. (2015). A guide to Bayesian model selection for ecologists. *Ecol. Monogr.*, 85, 3–28.

Johnson, M.F., Albertson, L.K., Algar, A.C., Dugdale, S.J., Edwards, P., England, J., *et al.* (2024). Rising water temperature in rivers: Ecological impacts and future resilience. *Wiley Interdiscip. Rev. Water*, 11, e1724.

Jonsson, Cohen & Carpenter. (2005). *Food Webs: From Connectivity to Energetics*. Elsevier.

Junker, J.R., Cross, W.F., Benstead, J.P., Huryn, A.D., Hood, J.M., Nelson, D., *et al.* (2020). Resource supply governs the apparent temperature dependence of animal production in stream ecosystems. *Ecol. Lett.*, 23, 1809–1819.

Kellner, K.F., Fowler, N.L., Petroelje, T.R., Kautz, T.M., Beyer Jr., D.E. & Belant, J.L. (2022). ubms: An R package for fitting hierarchical occupancy and N-mixture abundance models in a Bayesian framework. *Methods Ecol. Evol.*, 13, 577–584.

Mazurkiewicz, M., Górska, B., Renaud, P.E. & Włodarska-Kowalczuk, M. (2020). Latitudinal consistency of biomass size spectra - benthic resilience despite environmental, taxonomic and functional trait variability. *Sci. Rep.*, 10, 4164.

McNab, B.K. (2010). Geographic and temporal correlations of mammalian size reconsidered: a resource rule. *Oecologia*, 164, 13–23.

NEON. (2022). NEON 2022 (National Ecological Observatory Network). Fish electrofishing, gill netting, and fyke netting counts (DP1.20107.001).

NEON. (2023a). NEON. 2023. Continuous discharge (DP4.00130.001).

NEON. (2023b). NEON. 2023. Water quality (DP1.20288.001).

NEON. (2023c). NEON 2023 (National Ecological Observatory Network). Macroinvertebrate collection (DP1.20120.001), RELEASE-2024.

NEON. (2023d). NEON 2023 (National Ecological Observatory Network). Temperature (PRT) in surface water (DP1.20053.001).

Novak, B., Murry, B.A., Wesner, J.S., Gjoni, V., Junker, J., Shepta, E., *et al.* (2024). Threshold responses of freshwater fish community size spectra to invasive species. *Ecosphere*, 0, 1–12.

O’Gorman, E.J., Zhao, L., Pichler, D.E., Adams, G., Friberg, N., Rall, B.C., *et al.* (2017a). Unexpected changes in community size structure in a natural warming experiment. *Nat. Clim. Change*, 7, 659–663.

O’Gorman, E.J., Zhao, L., Pichler, D.E., Adams, G., Friberg, N., Rall, B.C., *et al.* (2017b). Unexpected Changes in Community Size Structure in a Natural Warming Experiment. *Nat. Clim. Change*, 7, 659–663.

Perkins, D.M., Durance, I., Edwards, F.K., Grey, J., Hildrew, A.G., Jackson, M., *et al.* (2018). Bending the rules: exploitation of allochthonous resources by a top-predator modifies size-abundance scaling in stream food webs. *Ecol. Lett.*, 21, 1771–1780.

Perkins, D.M., Perna, A., Adrian, R., Cermeño, P., Gaedke, U., Huete-Ortega, M., *et al.* (2019). Energetic equivalence underpins the size structure of tree and phytoplankton communities. *Nat. Commun.*, 10, 255.

Petchey, O.L. & Belgrano, A. (2010). Body-size distributions and size-spectra: universal indicators of ecological status? *Biol. Lett.*, 6, 434–437.

Pomeranz, J.P.F., Junker, J.R. & Wesner, J.S. (2022). Individual size distributions across North American streams vary with local temperature. *Glob. Change Biol.*, 28, 848–858.

Reuman, D.C., Mulder, C., Raffaelli, D. & Cohen, J.E. (2008). Three allometric relations of population density to body mass: theoretical integration and empirical tests in 149 food webs. *Ecol. Lett.*, 11, 1216–1228.

Royle, J.A., Dawson, D.K. & Bates, S. (2004). Modeling Abundance Effects in Distance Sampling. *Ecology*, 85, 1591–1597.

Saito, V.S., Perkins, D.M. & Kratina, P. (2021). A Metabolic Perspective of Stochastic Community Assembly. *Trends Ecol. Evol.*, 36, 280–283.

Song, C., Dodds, W.K., Rüegg, J., Argerich, A., Baker, C.L., Bowden, W.B., *et al.* (2018). Continental-scale decrease in net primary productivity in streams due to climate warming. *Nat. Geosci.*, 11, 415–420.

Stan Development Team. (2024). RStan: the R interface to Stan.

Virkar, Y. & Clauset, A. (2014). Power-law distributions in binned empirical data. *Ann. Appl. Stat.*, 8, 89–119.

Webster, J., Golladay, S., Benfield, E., D’Angelo, D. & Peters, G. (1990). Effects of forest disturbance on particulate organic matter budgets of small streams. *J. North Am. Benthol. Soc.*, 9, 120–140.

Wesner, J. & Pomeranz, J. (2023a). *isdbayes: Bayesian Hierarchical Modeling of Power Laws using brms*.

Wesner, J.S. & Pomeranz, J. (2023b). Isdbayes: Bayesian Hierarchical Modeling of Power Laws Using Brms.

Wesner, J.S., Pomeranz, J.P.F., Junker, J.R. & Gjoni, V. (n.d.). Bayesian hierarchical modelling of size spectra. *Methods Ecol. Evol.*, n/a.

West, G.B., Enquist, B.J. & Brown, J.H. (2009). A general quantitative theory of forest structure and dynamics. *Proc. Natl. Acad. Sci.*, 106, 7040–7045.

White, E.P., Ernest, S.K.M., Kerkhoff, A.J. & Enquist, B.J. (2007). Relationships between body size and abundance in ecology. *Trends Ecol. Evol.*, 22, 323–330.

Wood, S.N. (2017). *Generalized Additive Models: An Introduction with R, Second Edition*. 2nd edn. Chapman and Hall/CRC, Boca Raton.

Woodward, G., Ebenman, B., Emmerson, M., Montoya, J.M., Olesen, J.M., Valido, A., *et al.* (2005). Body size in ecological networks. *Trends Ecol. Evol.*, 20, 402–409.

Yule, Gilbert & Franz. (2020). Designing Biorepositories to Monitor Ecological and Evolutionary Responses to Change.

Yvon-Durocher, G., Montoya, J.M., Trimmer, M. & Woodward, G. (2011). Warming alters the size spectrum and shifts the distribution of biomass in freshwater ecosystems. *Glob. Change Biol.*, 17, 1681–1694.
